# Orthogonal ring-closing alkyne and olefin metathesis for the synthesis of small GTPase-targeting bicyclic peptides

**DOI:** 10.1038/ncomms11300

**Published:** 2016-04-14

**Authors:** Philipp M. Cromm, Sebastian Schaubach, Jochen Spiegel, Alois Fürstner, Tom N. Grossmann, Herbert Waldmann

**Affiliations:** 1 Department of Chemical Biology, Max-Planck-Institute of Molecular Physiology, Otto-Hahn-Strasse 11, D-44227 Dortmund, Germany; 2 Technische Universität Dortmund, Fakultät für Chemie and Chemische Biologie, Otto-Hahn-Strasse 6, D-44227 Dortmund, Germany; 3 Max-Planck-Institut für Kohlenforschung, Kaiser-Wilhelm-Platz 1, D-45470 Mülheim/Ruhr, Germany; 4 Chemical Genomics Centre of the Max Planck Society, Otto-Hahn-Strasse 15, D-44227 Dortmund, Germany; 5 Department of Chemistry and Pharmaceutical Sciences, VU University Amsterdam, De Boelelaan 1083, 1081 HV Amsterdam, The Netherlands

## Abstract

Bicyclic peptides are promising scaffolds for the development of inhibitors of biological targets that proved intractable by typical small molecules. So far, access to bioactive bicyclic peptide architectures is limited due to a lack of appropriate orthogonal ring-closing reactions. Here, we report chemically orthogonal ring-closing olefin (RCM) and alkyne metathesis (RCAM), which enable an efficient chemo- and regioselective synthesis of complex bicyclic peptide scaffolds with variable macrocycle geometries. We also demonstrate that the formed alkyne macrocycle can be functionalized subsequently. The orthogonal RCM/RCAM system was successfully used to evolve a monocyclic peptide inhibitor of the small GTPase Rab8 into a bicyclic ligand. This modified peptide shows the highest affinity for an activated Rab GTPase that has been reported so far. The RCM/RCAM-based formation of bicyclic peptides provides novel opportunities for the design of bioactive scaffolds suitable for the modulation of challenging protein targets.

## Abstract (web summary)

Bicyclic peptides can inhibit biological targets hard to address with small molecules. Here, the authors combine two orthogonal ring-closing reactions to produce bicyclic peptides with improved bioactivity thereby providing a strategy that can greatly improve the structural diversity of such peptides.

## Graphical abstract

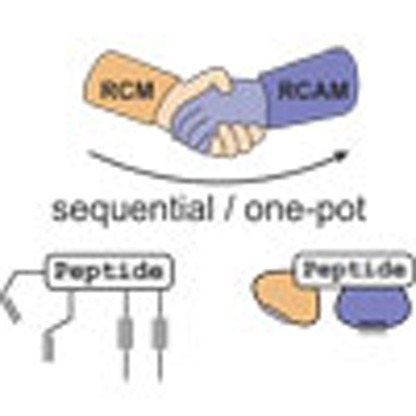

Macrocyclic peptides exhibit unique surface recognition properties and allow the stabilization of bioactive peptide conformations resulting in ligands with increased bioactivity and bioavailability^1^,^2^,^3^,^4^,^5^. Such scaffolds already proved useful for the modulation of biological targets which are intractable by typical small molecules, such as transcription factors and small GTPases^6^,^7^,^8^,^9^,^10^,^11^,^12^. Recently, structurally rigid bicyclic peptides obtained mainly by epitope grafting on disulfide-rich frameworks^13^,^14^ or by phage-display screening^15^,^16^ have emerged as particularly interesting inhibitor types. Given the potential of this new chemical modality as scaffold for next-generation therapeutics, the development of efficient synthetic methods that enable the introduction of non-natural fragments into peptidic bicycles is in high demand. This is particularly true for approaches that allow the design of scaffolds that go beyond the size of small epitopes.

For the synthesis of monocyclic peptides, a variety of methods is available^17^,^18^,^19^,^20^,^21^,^22^,^23^,^24^,^25^. Notably, in such peptides, the crosslink itself can directly contribute to bioactivity^26^,^27^,^28^. In this respect, hydrocarbon crosslinks formed by ruthenium-catalysed ring-closing olefin metathesis (RCM) proved particularly successful owing to the hydrophobic and inert character of those crosslinks^2^,^29^. Prominent examples involve hydrogen bond surrogates^8^,^30^,^31^ and hydrocarbon stapling^32^,^33^,^34^,^35^, which have provided a number of potent inhibitors of protein—protein interactions^2^. In these cases, the synthesis of bicyclic architectures requires the presence of multiple olefins, which causes selectivity problems during cyclization^36^,^37^. Undesired side reactions can be reduced by the selection of appropriate ring sizes and distances^38^, by functional group transformations^39^ and by tedious fine tuning of olefin reactivity^39^. Thus, only a small set of scaffolds is accessible by multiple RCM reactions^36^,^37^,^38^,^39^,^40^. This creates the need for synthesis methods that integrate two consecutive, chemically orthogonal metathesis reactions thereby enabling efficient chemo- and regioselective construction of complex bicyclic peptides. Ideally, such methods would be compatible with solid-phase peptide synthesis (SPPS).

Molybdenum-catalysed ring-closing alkyne metathesis^41^ (RCAM) shares many of the advantageous properties of RCM, and is in principle chemically orthogonal to ruthenium-catalysed RCM. RCAM has been applied for peptide macrocyclizations in solution^42^,^43^,^44^. However, the synthesis of bicyclic peptides by orthogonal ring-closing olefin and alkyne metathesis has not been explored so far. Here, we report the solid-phase synthesis of bicyclic peptides by means of orthogonal ring-closing olefin- and alkyne-metathesis reactions. We demonstrate that the alkyne macrocycle can be further functionalized selectively. The orthogonal RCM/RCAM system was successfully used to evolve a monocyclic peptide inhibitor of the small GTPase Rab8 into a bicyclic ligand with increased target affinity.

## Results

### RCAM and functionalization on solid support

**Figure 1:**
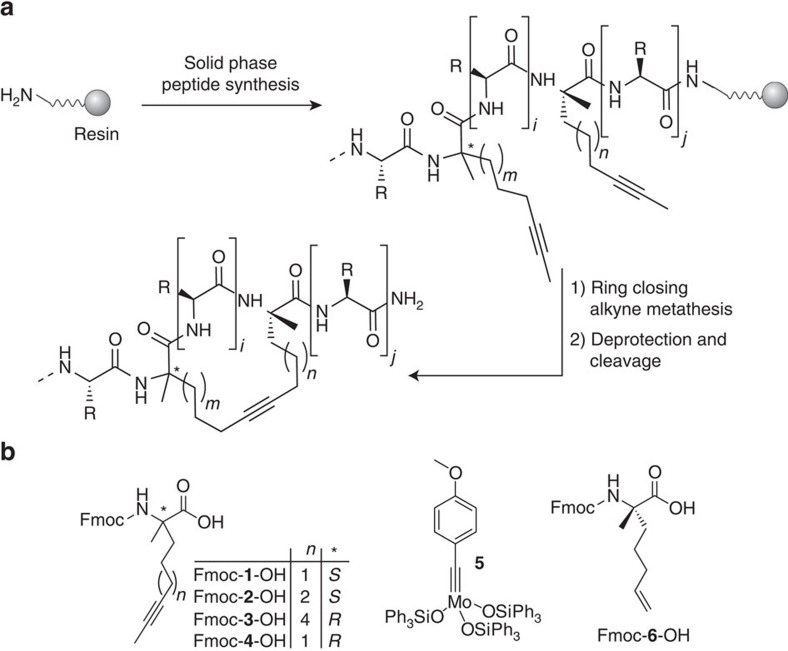
Alkyne macrocyclization. (**a**) The linear peptide is assembled via SPPS including the incorporation of two α-methylated-α-alkynylated building blocks (**1**–**4**). The C-terminal building block is always (*S*)-configured, the configuration of the N-terminal building block varies between the different architectures. Complex **5** is used to perform the RCAM reaction. (*i*=2, 3, 6, number of amino acids between non-natural building blocks; *j*=3, 6; *m*=1, 2, 4; *n*=1, 2; R=side chain of a proteinogenic amino acid) (**b**) Fluorenylmethoxycarbonyl (Fmoc) protected non-natural amino acids incorporated into the peptide sequence (alkyne: **1**–**4**, olefin: **6**). The alkyne building blocks **1**–**4** are either used in the (*S*)- or (*R*)-configuration depending on the macrocycle architecture. Mo-complex (**5**) used for RCAM.

**Figure 2:**
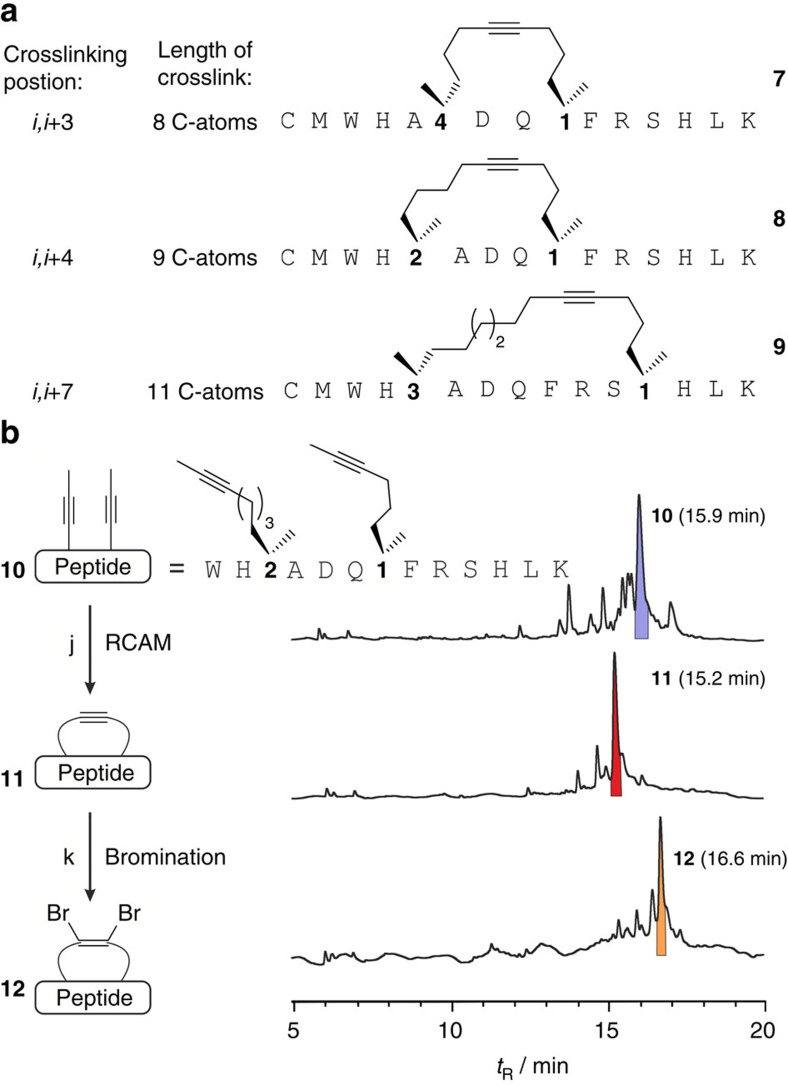
Ring-closing alkyne metathesis on solid support. (**a**) Sequences of peptides **7**–**9** containing different macrocylic architectures. (**b**) Sequence of peptide **10** with non-natural amino acids **1** and **2** at position *i* and *i*+4 (nine-carbon crosslink) with corresponding chromatograms of crude reaction mixtures before (**10**, top) and after RCAM (**11**, middle) and after dibromination (**12**, bottom). Corresponding product peaks are highlighted: open (**10**, blue), closed (**11**, red) and dibrominated (**12**, orange) macrocycle. Chromatograms were obtained after deprotection and release of intermediates from the resin. ‘j' represents Complex **5**, dry toluene, 40 °C, 2 × 1.5 h; ‘k' represents CuBr_2_, dry MeCN, 3 × 1 h.

To explore RCAM-based macrocyclization of peptides on solid support (Fig. 1a), two α-methyl-α-alkynyl building blocks (**1**–**4**) of varying linker length and configuration (Fig. 1b) were introduced into model peptides using Fmoc-based SPPS. Peptide sequences, architectures and relative spacing of non-natural amino acids (*i*,*i*+3, *i*,*i*+4 and *i*,*i*+7) were selected by analogy to previously explored RCM-based peptide macrocyclizations^33^. As proof-of-concept and to test the robustness of the reaction, we designed model peptides that contain all functionalities present among the 20 proteinogenic amino acids and yield macrocyclic peptides **7**–**9** after RCAM (Fig. 2a). Investigation of various RCAM conditions including the latest generation of stable Mo-complexes^45^,^46^ (Supplementary Table 1) revealed efficient conversions after 3 h at 40 °C in toluene if Tentagel rink amide resin and complex **5** (Fig. 1a) were used (Supplementary Figs 2–5, Supplementary Table 2). Under these conditions, macrocycles were formed for all three architectures (**7**–**9**) with the best results obtained for an *i*,*i*+4 geometry and a final crosslink of nine carbon atoms (**8**). Shortening the hydrocarbon bridge from nine to eight carbon atoms reduces the efficiency of the reaction presumably due to increased ring strain (Supplementary Fig. 4).

Selective functionalization of the alkyne linker embedded in the macrocycle was achieved, after treatment of macrocyclic peptide **11** with CuBr_2_ in dry acetonitrile on solid support to yield dibrominated olefin **12** (Fig. 2b). Notably, the reaction can be performed conveniently with different resin-bound peptides (for all tested architectures: *i*,*i*+3, *i*,*i*+4 and *i*,*i*+7) using standard syringe reactors (Supplementary Figs 6–8, Supplementary Table 2). Full conversion in the dibromination reaction is only achieved after multiple treatments with CuBr_2_ and for peptides that lack the two N-terminal sulfur containing amino acids (Cys and Met).

### Bicyclic peptide synthesis via orthogonal RCM and RCAM

**Figure 3:**
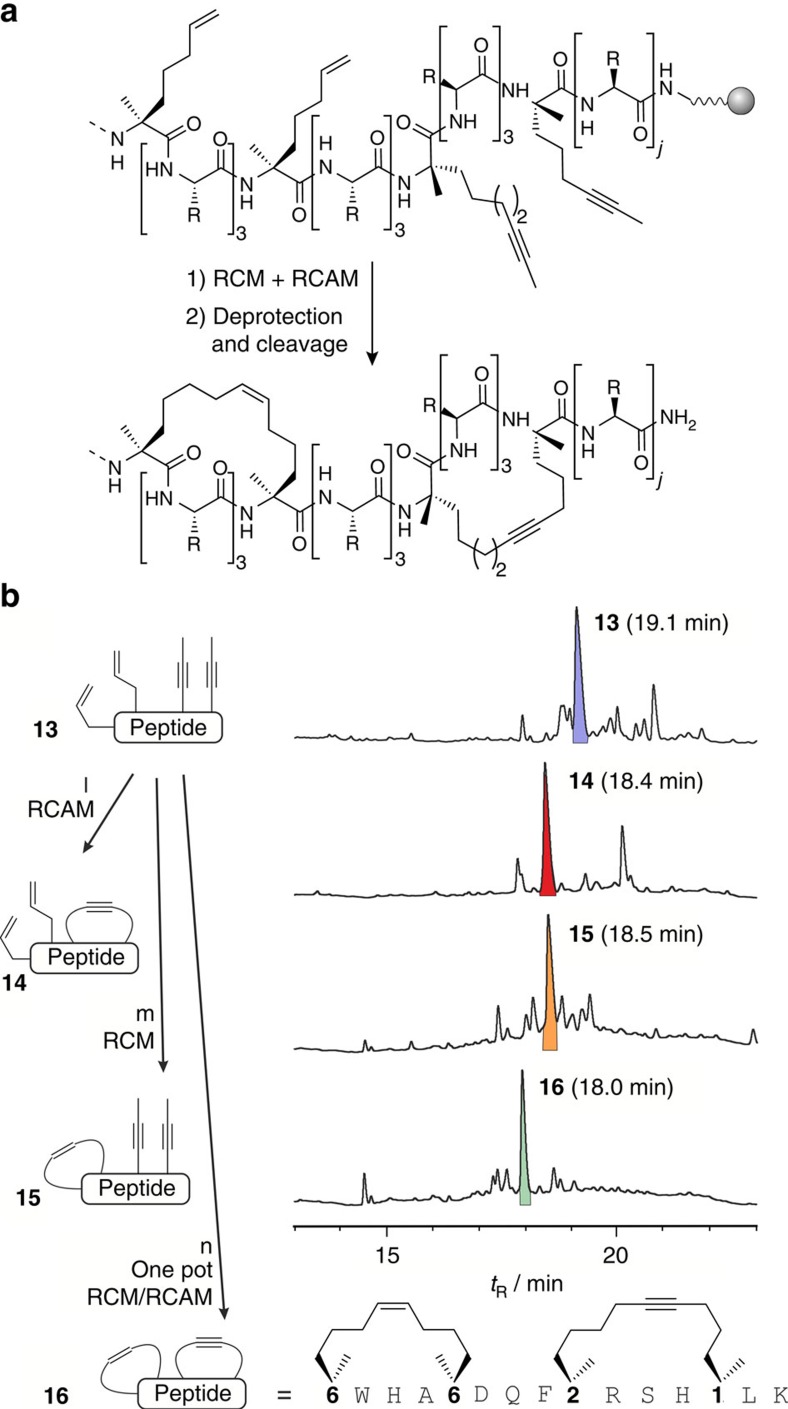
Solid phase synthesis of bicyclic peptides by means of the RCM/RCAM method. (**a**) General scheme for the synthesis of bicyclic peptides obtained by means of RCM and RCAM of the acyclic precursor peptides. (*j*=2, number of C-terminal amino acids; R=side chain of a proteinogenic amino acid except Cys or Met). (**b**) Sequence of bicyclic test peptide **16** bearing an *i*,*i*+4 olefin crosslink (eight C-atoms) and an *i*,*i*+4 alkyne crosslink (nine C-atoms). Chromatograms of crude reaction mixtures of peptide **16** before macrocyclization (**13**, top), after RCAM (**14**, second) and RCM (**15**, third), respectively, and after simultaneous (one-pot) RCM and RCAM (**16**, bottom). Corresponding product peaks are highlighted: fully open (**13**, blue), alkyne monocycle (**14**, red), olefin monocycle (**15**, orange) and bicyclic peptide (**16**, green). Chromatograms were obtained after deprotection and cleavage of resin-bound intermediates. ‘l' represents Complex **5**, dry toluene, 40 °C, 2 × 1.5 h; ‘m' represents Grubbs first-generation catalyst, DCE, 3 × 2 h; ‘n' represents Complex **5**, Grubbs first-generation catalyst, dry toluene, 40 °C, 2 × 1.5 h.

To determine whether RCM and RCAM can be performed orthogonally within one peptide sequence (Fig. 3a), peptide **16** was synthesized which embodies two alkyne-functionalized building blocks (**1** and **2**) in *i*,*i*+4-position at the carboxy (C) terminus, and two olefin-containing amino acids (**6**, Fig. 1b) in *i*,*i*+4-position at the amino (N) terminus (Fig. 3b). In peptide **16**, an olefin macrocycle can be formed next to an alkyne-bearing macrocycle (Fig. 3b). The treatment of immobilized precursor peptide **13** (blue peak) with either complex **5** or Grubbs first-generation catalyst leads to selective formation of the alkyne (**14**, red peak) and olefin macrocycle (**15**, orange peak), respectively (Fig. 3b, Supplementary Figs 9 and 10). HPLC-MS analyses of the alkyne and olefin crosslinked intermediates (**14** and **15**) reveal highly selective formation of the desired macrocycle without formation of an alternative cyclization product (Supplementary Fig. 10). Both monocycles can be converted into the bicyclic product **16** by means of the second metathesis reaction. This result is remarkable since previous attempts of orthogonal macrocycle formation within peptides failed^44^, but were successful only for the assembly of simple building blocks^47^. In an even more demanding set-up, the simultaneous closure of both macrocycles in a one-pot reaction was tested (instead of the previous sequential synthesis). Strikingly, treatment of the open peptide precursor **13** with a mixture of complex **5** and Grubbs first-generation catalyst also yields the desired bicyclic peptide **16** (green peak, Fig. 3b and Supplementary Fig. 10).

**Figure 4:**
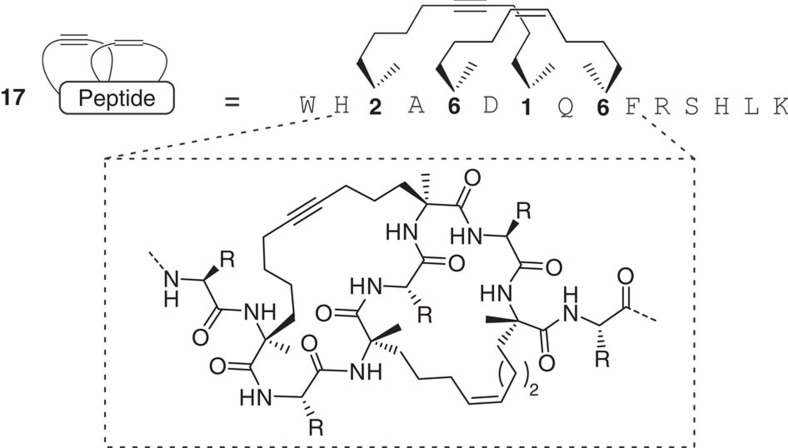
Sequence and chemical structure of the engulfed bicyclic peptide 17. R, side chain of a proteinogenic amino acid except Cys or Met.

In peptide **16**, the two individual macrocycles are sequentially arranged along the amino acid chain, that is, the two individual macrocycles are linked by a linear amino acid sequence. A synthetically more challenging setup involves the synthesis of two entangled macrocyles resulting in more constrained peptide scaffolds (for example, peptide **17**, Fig. 4). In this architecture, an edge-on bimacrocycle structure is generated as opposed to a linear macrocycle arrangement as in peptide **16**. Most notably, entangled bicyclic peptide **17** (Fig. 4) was also efficiently formed in both the sequential as well as the one-pot synthesis (Supplementary Figs 11 and 12).

### Bicyclic ligands of the small GTPase Rab8

To demonstrate the potential of this robust orthogonal RCM/RCAM macrocyclization, we aimed at the improvement of a monocyclic bioactive peptide targeting a challenging protein. Small GTPases comprise a protein superfamily with clinically highly relevant, yet particularly challenging, drug targets^48^,^49^,^50^,^51^,^52^,^53^,^54^. Despite enormous efforts, no efficient inhibitors of small GTPases have reached clinical trials. Importantly, the target affinity of small molecule modulators typically does not exceed the low-to-medium micromolar range^50^,^51^. Among the small GTPase superfamily, Rab proteins (Ras-related in brain) constitute key regulators of intracellular vesicular transport and trafficking^55^,^56^. As starting point for the generation of a bicyclic peptide, we selected the hydrocarbon-stapled monocyclic peptide **StRIP3**, which binds the small GTPase Rab8a with moderate affinity (dissociation constant (*K*_d_)=20.7 μM, Table 1, entry 2)^12^. **StRIP3** resembles the only known inhibitor of a Rab protein–protein interaction^12^ and is based on the interaction motif of Rab6-interacting protein 1 (**wt-R6IP**, Table 1, entry 1)^57^. Initially, alkyne-bearing macrocycles based on **StRIP3** were explored in which the *i*,*i*+4 olefin crosslink was replaced by alkynes with varying crosslink length (9–10 carbon atoms, Table 1, entry 3–5). The 10-carbon crosslink requires double incorporation of building block **2** (entry 3). A nine-carbon crosslink is generated by incorporation of two different building blocks (**1** and **2**), which results in two different architectures (entry 4: **1**/**2**, entry 5: **2**/**1**). The synthesis of an eight-carbon crosslinked **StRIP3** derivative containing building block **1** twice was not possible, most likely due to high ring strain caused by the linear geometry of the triple bond.

**Figure 5:**
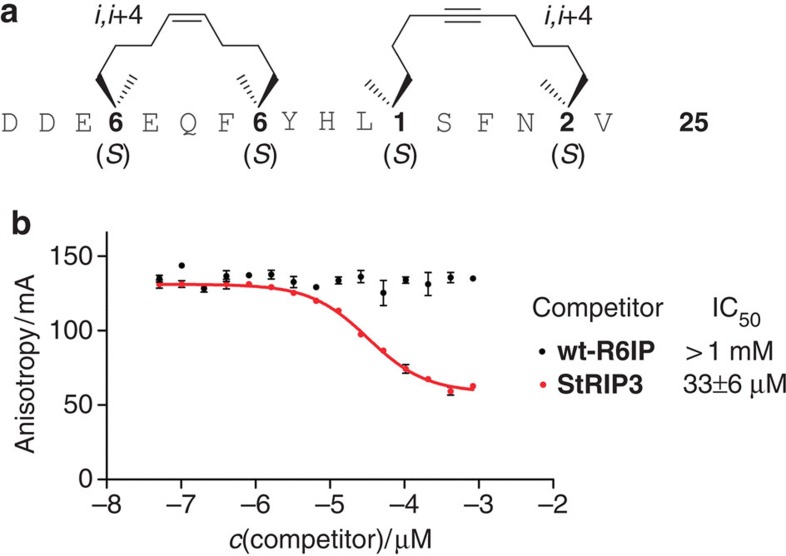
Sequence and binding studies of peptide 25. (**a**) Sequence of bicyclic peptide **25** showing highest affinity for Rab8a_6–176_(GppNHp). (**b**) Competition of fluorescein-labelled peptide **25** (60 nM) bound to Rab8a(GppNHp; 15 μM) with increasing concentrations of acetylated peptides **StRIP3** and **wt-R6IP** (competitors). Errors represent 1σ of triplicates.

In addition, dibrominated (entry 6–8) and bicyclic peptides (entry 9–14) were synthesized resulting in a total of 12 **StRIP3** derivatives grouped into four subfamilies (Table 1, Supplementary Table 3): (i) alkyne mono-macrocyclic peptides **18**–**20** (entry 3–5); (ii) dibrominated olefin macrocyclic peptides **21**–**23** (entry 6–8); (iii) orthogonally macrocyclized peptides **24**–**26** carrying the original olefin crosslink and an additional alkyne crosslink at the C terminus (entry 9–11); and (iv) bicyclic peptides **27**–**29** with exchanged positions for the alkyne and the olefin crosslink (entry 12–14). Since the N-terminal part of parent peptide **StRIP3** is already constrained by the olefin macrocycle, we aimed for the introduction of a new macrocycle in the C-terminal part. We reasoned that additional constraint could further stabilize the bioactive peptide conformation. Owing to a lack of structural information, it is not obvious which amino acids are directly involved in Rab-binding. For this reason, we selected two amino acids with hydrophobic side chains (L911 and A915) for macrocycle introduction as their non-polar side chains are potentially mimicked by the hydrocarbon macrocycle. All the peptides were synthesized via SPPS and modified with an N-terminal fluorescein–polyethyleneglycol label (Supplementary Table 3) to enable determination of their binding affinity towards activated Rab8a_6-176_(GppNHp) in a fluorescence polarization (FP) assay (Supplementary Fig. 13). After initial ranking of the peptides by means of relative *K*_d_ values (rel. *K*_d_, Table 1, Supplementary Table 4), the affinity of the best binders (peptide **21**, **25** and **28**) was determined in an independent FP assay run in triplicates (Table 1, Supplementary Fig. 14). Replacement of the olefin by an alkyne crosslink yields peptides **18**–**20** with affinities comparable to **StRIP3**. In contrast, the dibrominated olefin derivatives **21**–**23** show improved affinity towards Rab8a_6–176_ with peptide **21** being the most potent binder within this subfamily (*K*_d_=10.7 μM, Table 1). Peptide **21** shows a 2-fold increased binding affinity when compared with **StRIP3**. Notably, an even higher improvement in binding affinity to Rab8a_6–176_(GppNHp) is observed for two of the bicyclic peptides **24**–**29**, namely peptide **25** and **28** (Table 1 and Supplementary Table 4). In both the cases, the nine-carbon alkyne crosslink (with **1** at N-terminal and **2** at C-terminal position within the sequence) provides the most potent architecture resulting in two significantly improved ligands for activated Rab8a_6–176_ (*K*_d_[**25**]=6.6 μM; *K*_d_[**28**]=9.6 μM). Bicyclic peptide **25** (Fig. 5a) is more than three times more potent than the parent hydrocarbon stapled peptide **StRIP3** and displays a more than 15-fold increased binding affinity compared with the unmodified wild-type peptide **wt-R6IP**. Binding affinity of peptide **25** was confirmed in microscale thermophoresis measurements. On the basis of fluorescence intensity, an affinity for Rab8a_6–176_(GppNHp) was observed (*K*_d_[**25**]=11 μM, Supplementary Table 5, Supplementary Figs 15 and 16), which is in the range of our FP measurements (*K*_d_[**25**]=6.6 μM, see above). In addition, FP competition experiments were performed using a complex between labelled peptide **25** and Rab8a_6–176_(GppNHp), which was treated with an excess of acetylated **StRIP3**. In this setup, we observed full displacement of peptide **25** (IC_50_=33 μM, red Fig. 5b). As one would expect, the acetylated low-affinity peptide **wt-R6IP** does not compete with peptide **25** (black, Fig. 5b). These results verify reversible binding of peptide **25** to the same site on Rab8 as parent peptide **StRIP3**.

## Discussion

We identified conditions that enable the performance of RCAM reactions in conjunction with SPPS allowing alkyne-based macrocyclization of peptide sequences involving all natural side-chain functionalities. Subsequent functionalization of the alkyne allows further modification of the macrocycle, which opens new perspectives in the design of macrocyclic scaffolds. In addition, we report the chemo- and regioselective synthesis of bicyclic peptides bearing an alkyne as well as an olefin crosslink accessible via orthogonal ring-closing olefin and alkyne metathesis on solid support. This approach allows direct control of the individual macrocyclization reaction and enables the formation of bicyclic peptides with novel architectures combining two different macrocycles within the same peptide sequence. The applicability of such scaffolds for highly challenging targets, such as small GTPases, was demonstrated via identification of the currently most potent binder of an activated Rab GTPase (peptide **25**, *K*_d_=6.6 μM). Since the bioactivity of a peptide is mainly determined by its secondary structure^1^,^2^, alkyne macrocyclization and the orthogonal introduction of bicyclic alkyne/olefin macrocycles within the same peptide sequence give rise to novel constrained peptide architectures with high potential for the targeting of currently intractable proteins.

## Methods

### General

For abbreviations and detailed information about the experimental procedures, analytical data and FP binding curves, see Supplementary Figs 1–16, Supplementary Tables 1–5, Supplementary Note 1 and Supplementary Methods.

### Synthesis of building blocks **1**–**4**

Synthesis of the Fmoc protected building blocks **1**–**4** was performed according to adapted protocols using Ni(II)-BPB ((*R/S*)-2-[N-(N′-benzylpropyl)amino]benzophenone) complexes^58^,^59^. For a detailed description of building block synthesis and analytical data, see Supplementary Methods.

### Peptide synthesis

Peptides were synthesized according to standard Fmoc-chemistry for SPPS using HCTU (*O*-(6-chlorobenzotriazol-1-yl)-*N*,*N*,*N*′,*N*′-tetramethyluronium hexafluorophosphate) and COMU (1-[(1-(cyano-2-ethoxy-2-oxoethylidenaminooxy)-dimethylaminomorpholino)]-uronium hexafluorophosphate) as coupling reagents (Supplementary Fig. 1). For more detailed information about peptide synthesis, see Supplementary Methods and Supplementary Tables 2 and 3.

### Ring-closing alkyne metathesis

The dried resin was swollen and shrunken under argon alternating in dry diethyl ether and dry toluene (3 × each). Afterwards 0.5 ml of a solution of the alkyne-metathesis complex **5** (2 mg ml^−1^) in dry toluene was added and the reaction mixture was stirred at 40 °C for 1.5 h. During the reaction time, argon was bubbled through the reaction mixture to evaporate the 2-butyne. After addition of 0.5 ml of fresh complex **5** solution, the mixture was stirred at 40 °C for 1.5 h.

### Ring-closing olefin metathesis

The dried resin was swollen in 1,2-dichloroethane (DCE) for 15 min. Subsequently, 0.5 ml of a solution of Grubbs first-generation catalyst (2 mg ml^−1^) in DCE was added to the resin and reacted for 2 h at room temperature. During the reaction time, argon was bubbled through the reaction mixture to remove ethene. The procedure was repeated twice.

### One-pot ring-closing alkyne and olefin metathesis

The dried resin was swollen and shrunken under argon alternating in dry diethyl ether and dry toluene (3 × each). Afterwards 0.5 ml of a solution of the alkyne-metathesis complex **5** (2 mg ml^−1^) and Grubbs first-generation catalyst (2 mg ml^−1^) in dry toluene was added and the reaction mixture stirred at 40 °C for 1.5 h. During the reaction time, argon was bubbled through the reaction mixture to evaporate 2-butyne and ethene. After the addition of 0.5 ml of fresh complex solution (alkyne complex **5** and Grubbs first-generation catalyst), the mixture was stirred at 40 °C for 1.5 h.

### Dibromination of alkyne macrocycles

The dried resin was swollen in dry MeCN for 15 min and treated with a mixture of CuBr_2_ in dry MeCN (2 mg ml^−1^) for 2 h. The reaction was performed in a Syringe reactor and the procedure was repeated twice.

### Protein expression and purification

The expression and purification of Rab8a_6–176_ was performed by analogy to full-length Rab8a according to established protocols^60^,^61^.

### Nucleotide exchange

Nucleotide exchange was performed according to previously established protocols^60^,^62^. Briefly, for nucleotide removal, Mg^2+^ was removed by the addition of a 5-fold excess of EDTA and reacted for 1 h at room temperature. The protein solution was desalted using a PD-10 desalting column Sephadex G-25 DNA Grade (GE Healthcare) with elution buffer consisting of 20 mM HEPES (pH 7.5), 50 mM NaCl, 1 mM TCEP. After removal of Mg^2+^, the protein was diluted to 80–100 μM before the addition of ZnCl_2_ (500 μM) and (NH_4_)_2_SO_4_ (200 mM). After the addition of alkaline phosphatase (5 U mg^−1^Rab protein), the mixture was incubated for 16 h at 4 °C. For nucleotide exchange, the mixture contained a 5-fold excess of GppNHp during alkaline phosphatase incubation. Afterwards, the mixture was desalted using a PD-10 desalting column Sephadex G-25 DNA Grade (GE Healthcare) with elution buffer consisting of 25 mM HEPES (pH 7.5) 150 mM NaCl, 1 mM TCEP, 1 mM MgCl_2_ and 1 μM GppNHp.

### Fluorescence polarization assay

Rab8a_6–176_(GppNHp) was serially diluted in a buffer containing 25 mM HEPES (pH 7.5), 150 mM NaCl, 1 mM MgCl_2_, 1 mM TCEP, 0.01% Tween 20 and 1 μM GppNHp, treated with 66 nM fluorescein-labelled peptides and incubated for 4 h at room temperature. Fluorescence polarization values (*λ*_ex_=470 nm, *λ*_em_=525 nm) were determined at room temperature. Initial studies for alkyne macrocyclized peptides were performed as single measurements. Final affinity measurements of a subset of peptides were performed in triplicates. After correction for changes in fluorescence intensity upon binding, the fluorescence anisotoropy data were converted into fraction bound of the FITC-labelled peptide and fitted to a one-site binding model derived from the law of mass action using *K*_d_ as the only fitting parameter (for details, see Supplementary Methods)^63^. Nonlinear regression was performed in Prism 5.0 (Graphpad)^64^.

### Competition fluorescence polarization assay

Acetylated peptides were serially diluted and incubated with a mixture of the fluorescein-labelled peptide and Rab8a_6–176_ (GppNHp) at room temperature for 1 h. Fluorescence polarization was determined and IC_50_ values were calculated by nonlinear regression analysis using Prism 5.0 software (GraphPad)^64^.

## Additional information

**How to cite this article:** Cromm, P. M. *et al*. Orthogonal ring-closing alkyne and olefin metathesis for the synthesis of small GTPase-targeting bicyclic peptides. *Nat. Commun.* 7:11300 doi: 10.1038/ncomms11300 (2016).

## Supplementary Material

Supplementary Information Supplementary Figures 1-16, Supplementary Tables 1-5, Supplementary Note 1, Supplementary Methods and Supplementary References

## Figures and Tables

**Table 1:**
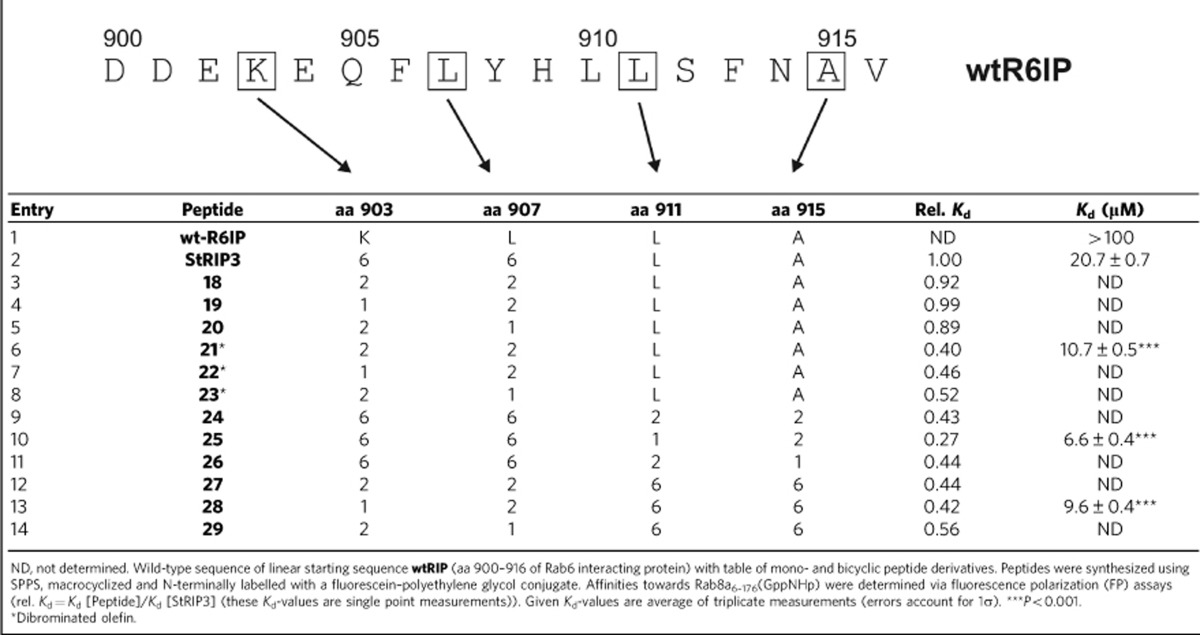
Small GTPase targeting peptides.
