# Supplementary material for: Orthogonal ring-closing alkyne and olefin metathesis for the synthesis of small GTPase-targeting bicyclic peptides
Source: Nat Commun. 2016 Apr 14;7:11300. doi: 10.1038/ncomms11300 (PMC4834642; doi:10.1038/ncomms11300)
Supplement: Supplementary Information — Supplementary Figures 1-16, Supplementary Tables 1-5, Supplementary Note 1, Supplementary Methods and Supplementary References [file ncomms11300-s1.pdf]

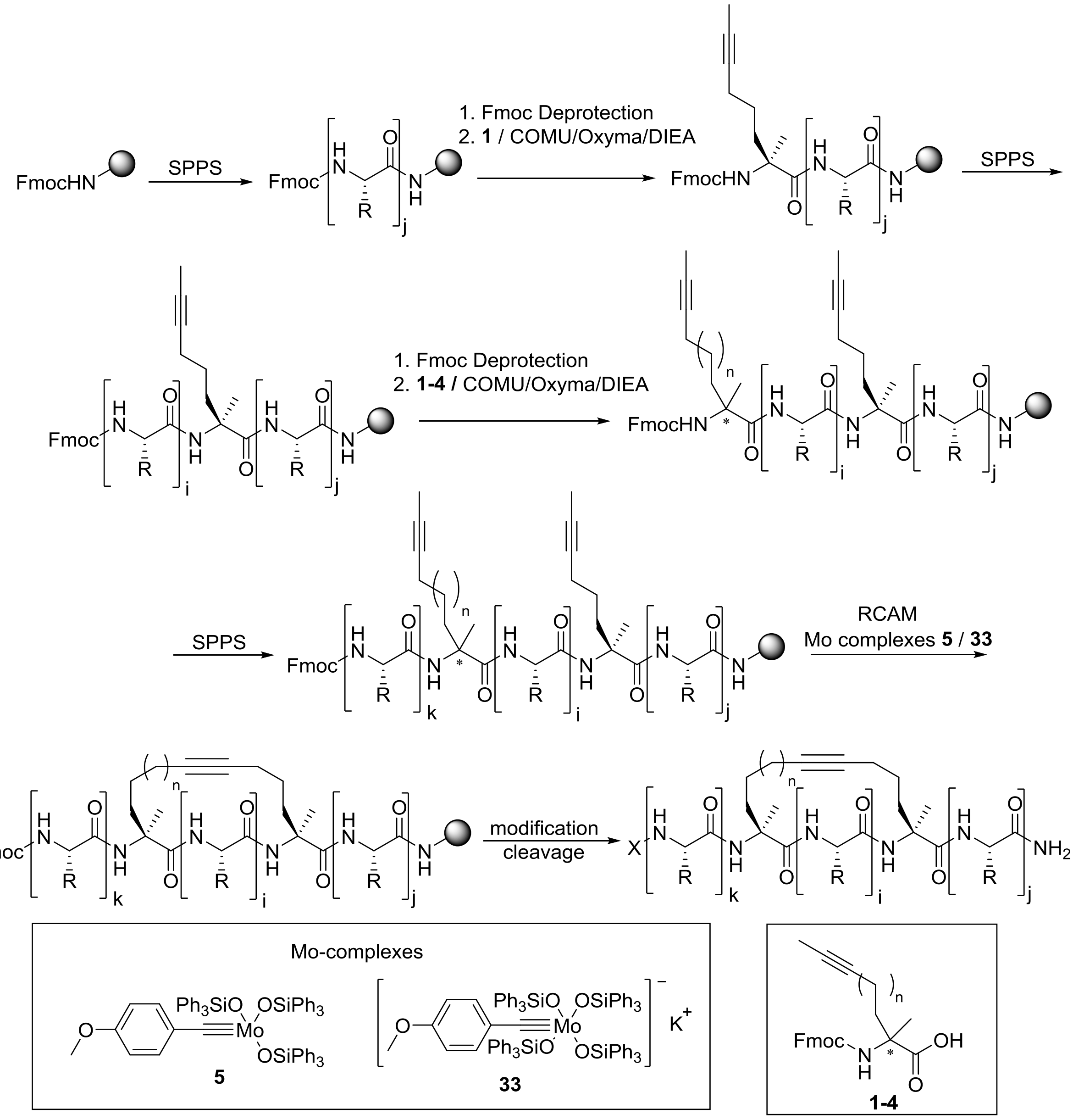

Supplementary Figure 1. Synthesis of alkyne macrocyclized peptides. The metathesis reaction was optimized using different resins and reaction conditions (Supplementary Table 1). A detailed overview of all synthesized peptides (sequences, analytical data) is shown in Supplementary Table 2 and Supplementary Table 3. j = number of C-terminal amino acids (2, 6); i = number of amino acids between the building blocks (2, 3, 6); k = number of N-terminal amino acids after the last building block (2, 4); n = 1, 2, 4; X = Fmoc, FITC

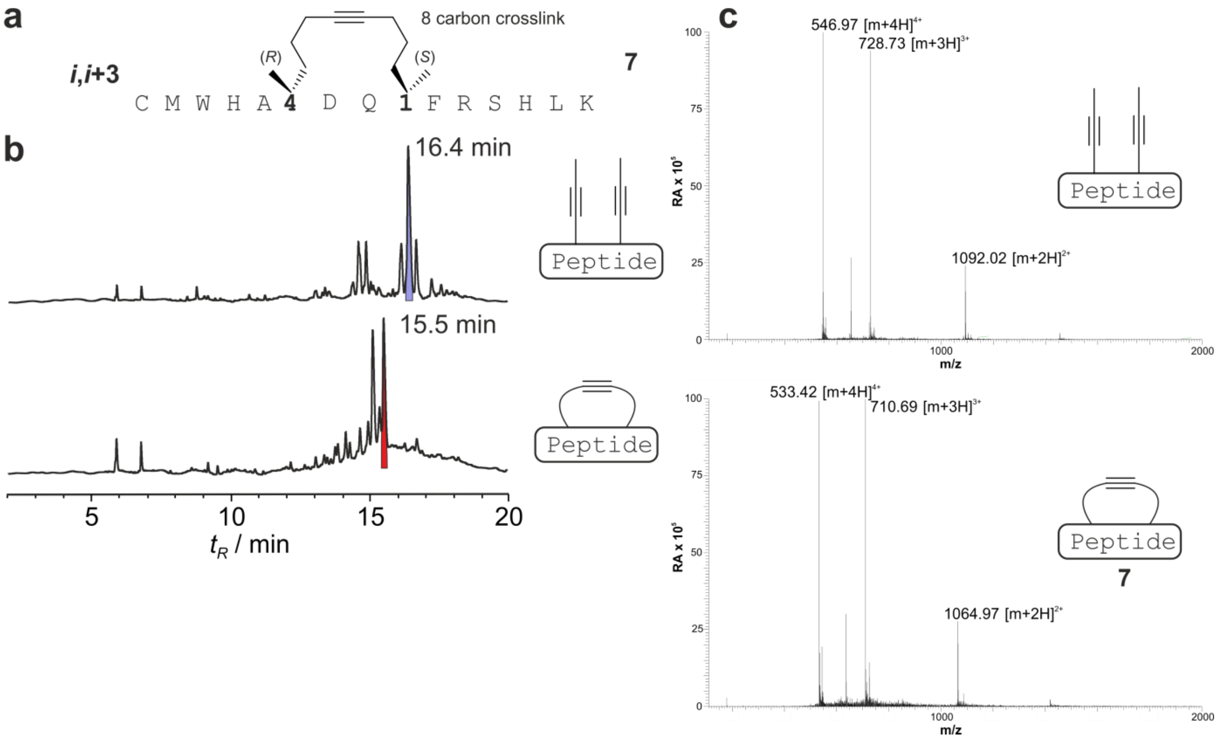

Supplementary Figure 2. (a) Sequence of the *i*,*i*+3 macrocyclized peptide **7** containing N-terminal Cys and Met residues. (b) HPLC traces of **7** in the open- (top) and closed conformation (bottom).The product peak is highlighted accordingly. (c) Mass spectra of the open- (top) and closed (bottom) alkyne crosslink. The HPLC chromatograms are taken from crude reaction mixtures without any further purification.

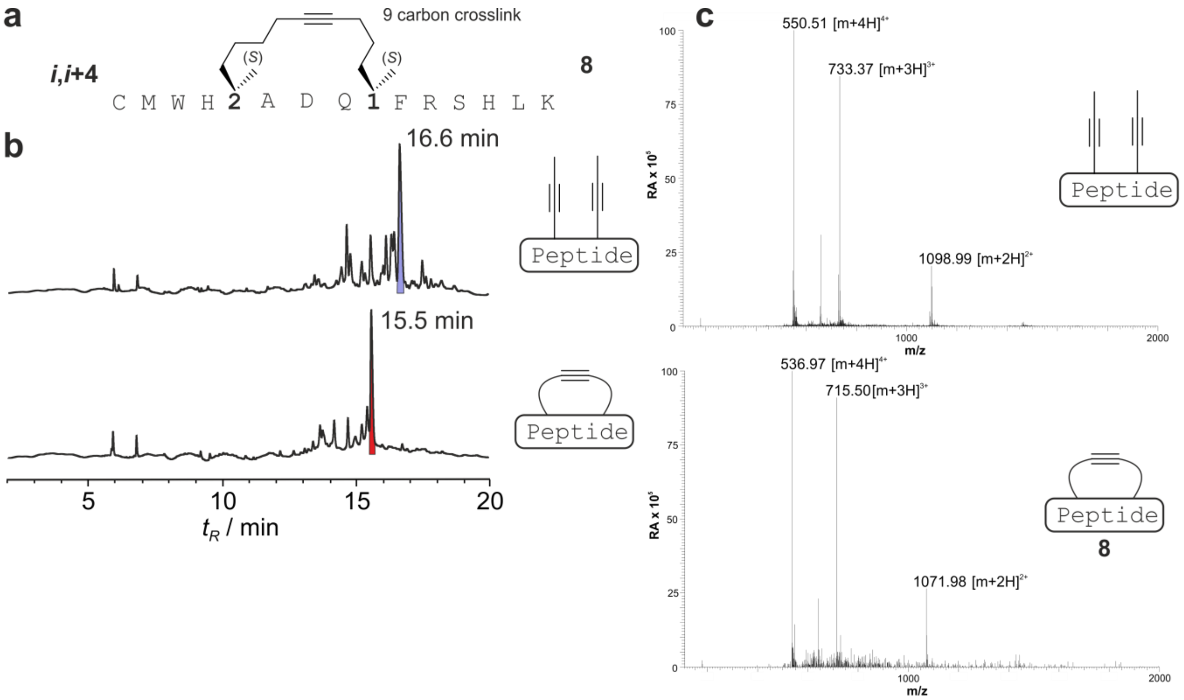

Supplementary Figure 3. (a) Sequence of the *i*,*i*+4 macrocyclized test peptide **8** containing N-terminal Cys and Met residues. (b) HPLC traces of **8** in the open- (top) and closed conformation (bottom).The product peak is highlighted accordingly. (c) Mass spectra of the open- (top) and closed (bottom) alkyne crosslink. The HPLC chromatograms are taken from crude reaction mixtures without any further purification.

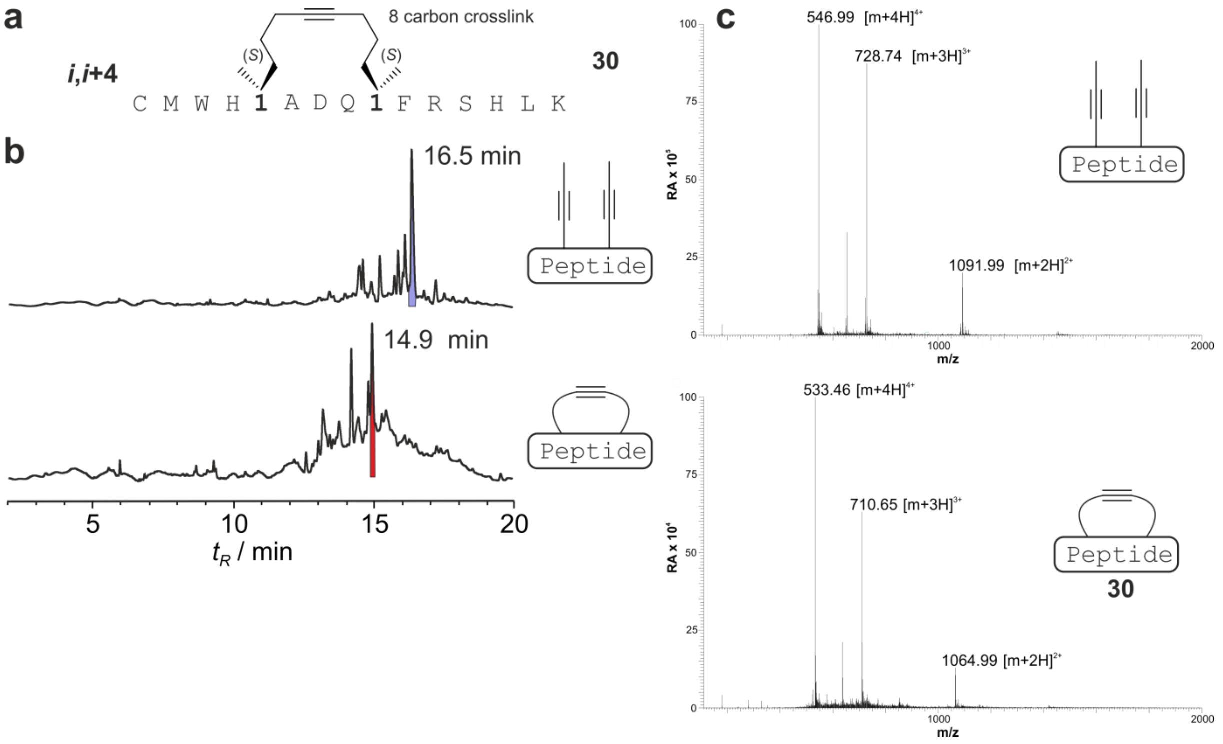

Supplementary Figure 4. (a) Sequence of the *i*,*i*+4 macrocyclized test peptide **30** containing N-terminal Cys and Met residues. The reduced size of the macrocycle (8 carbon atoms) decreases metathesis efficacy. (b) HPLC traces of **30** in the open- (top) and closed conformation (bottom).The product peak is highlighted accordingly. (c) Mass spectra of the open- (top) and closed (bottom) alkyne crosslink. The HPLC chromatograms are taken from crude reaction mixtures without any further purification.

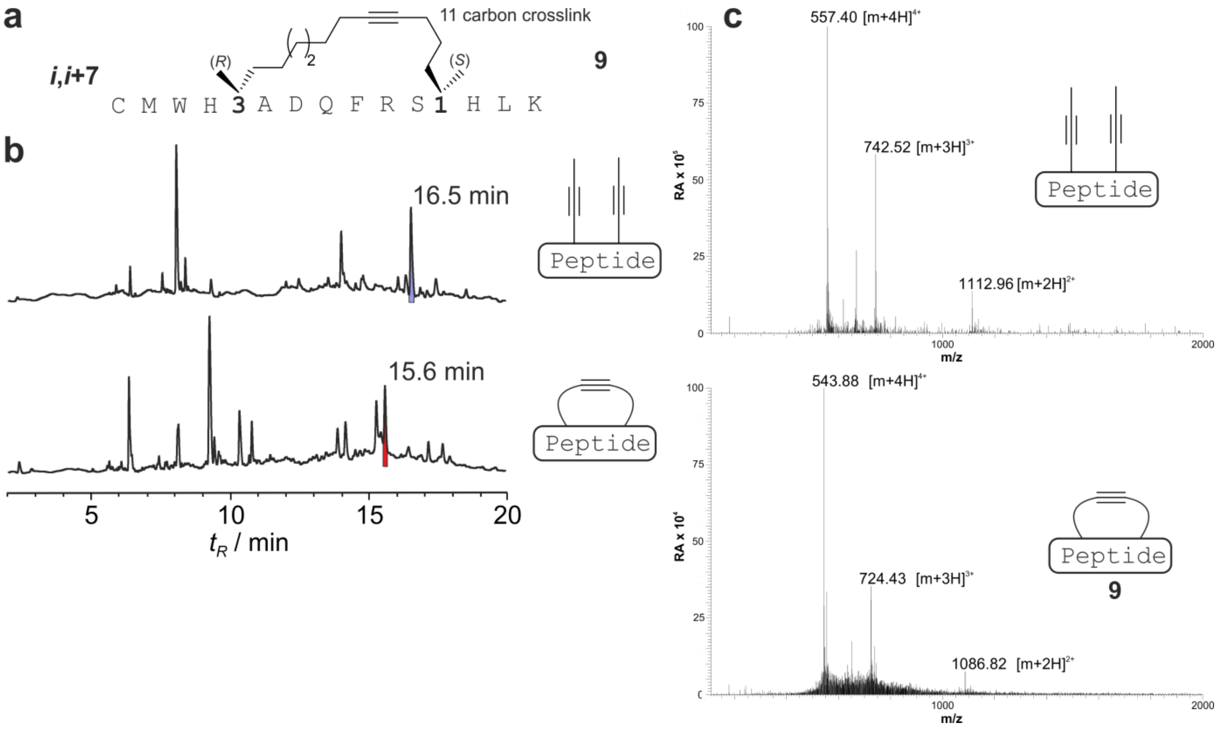

Supplementary Figure 5. (a) Sequence of the *i*,*i*+7 macrocyclized test peptide **9** containing N-terminal Cys and Met residues. (b) HPLC traces of **9** in the open- (top) and closed conformation (bottom).The product peak is highlighted accordingly. (c) Mass spectra of the open- (top) and closed (bottom) alkyne crosslink. The HPLC chromatograms are taken from crude reaction mixtures without any further purification.

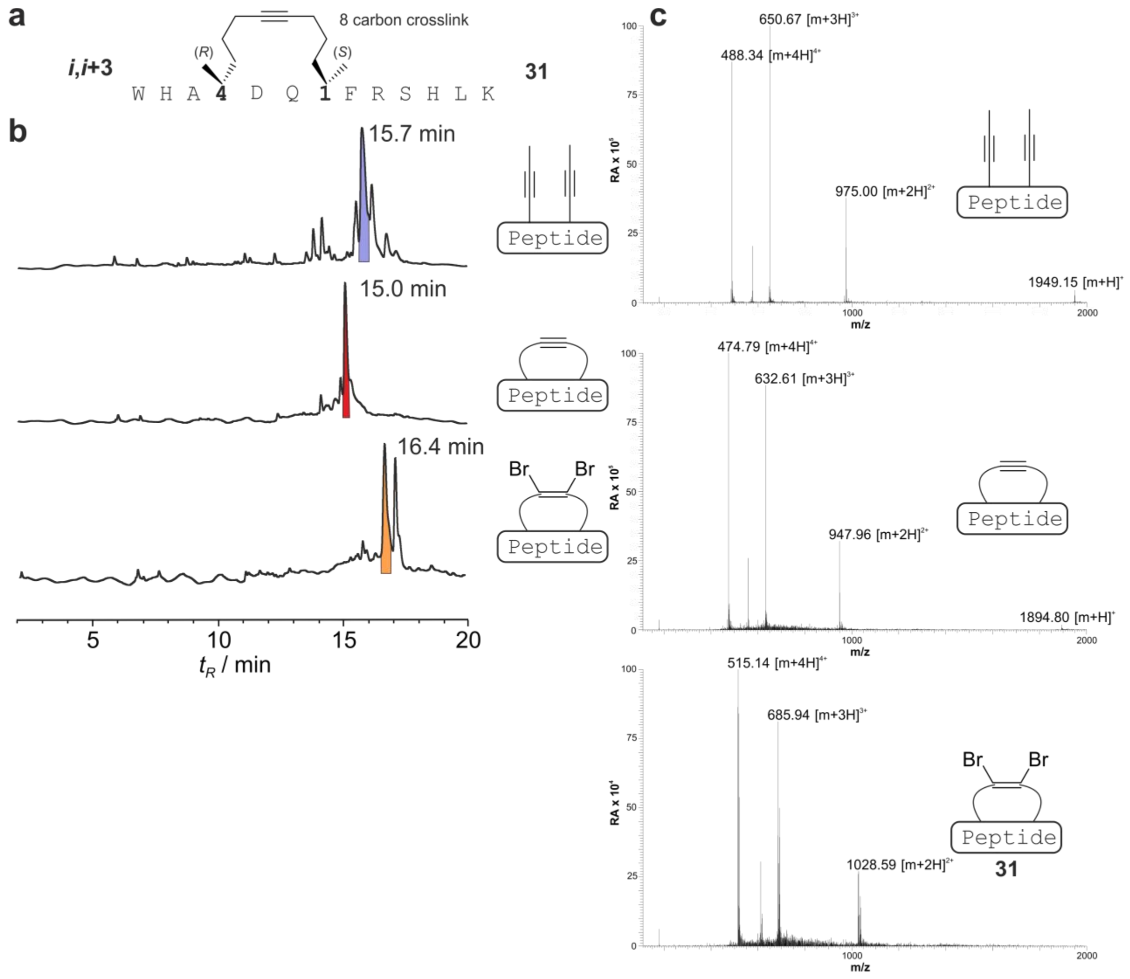

Supplementary Figure 6. (a) Sequence of the *i*,*i*+3 macrocyclized test peptide **31** without the N-terminal Cys and Met residues. After RCAM the alkyne macrocycle is dibrominated using $CuBr_2$. (b) HPLC traces of **31** in the open- (top), closed- (middle) and dibrominated conformation (bottom).The product peak is highlighted accordingly. (c) Mass spectra of the open- (top) and closed (middle) alkyne crosslink and the bibrominated olefin (bottom). The HPLC chromatograms are taken from crude reaction mixtures without any further purification.

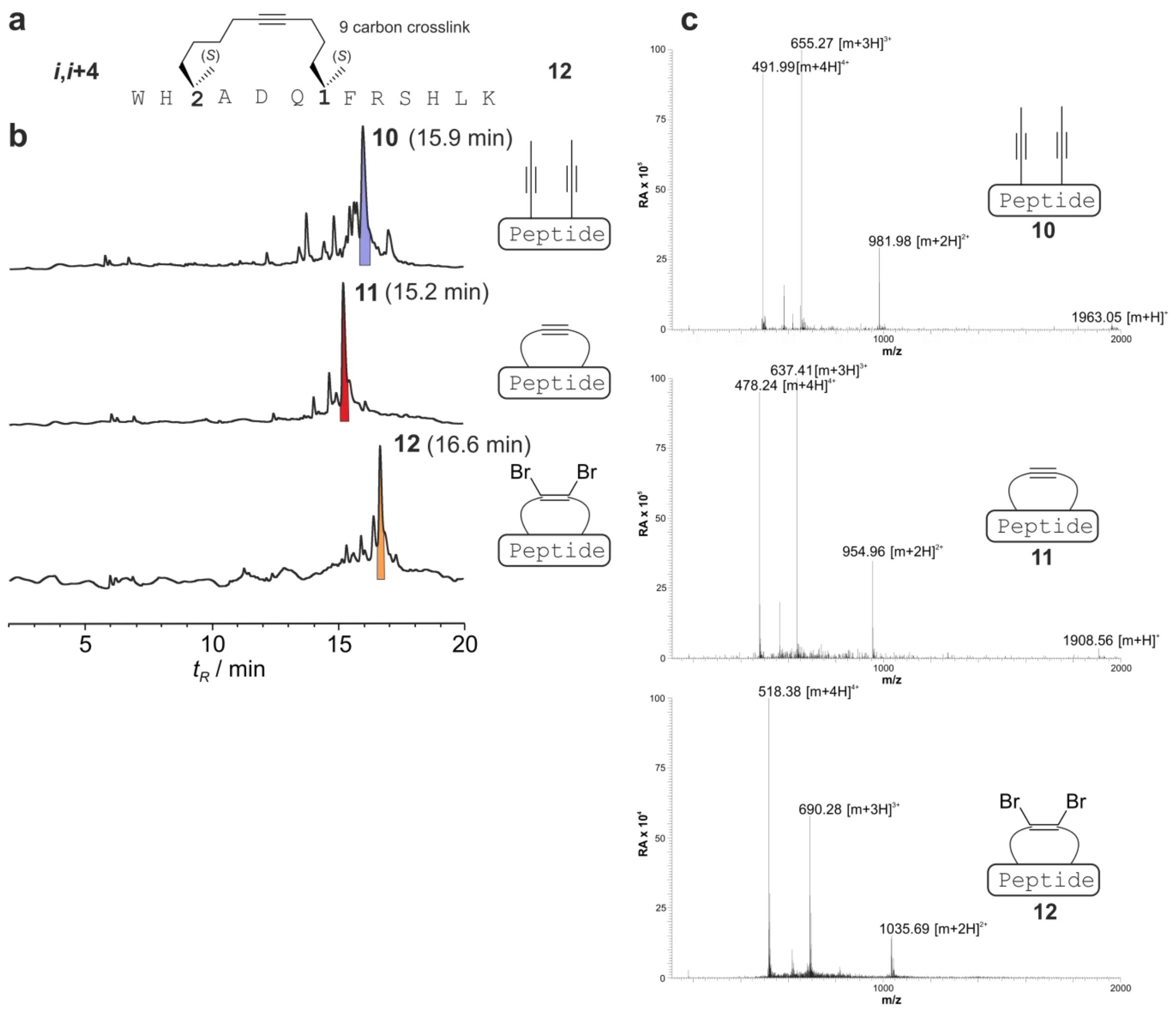

Supplementary Figure 7. (a) Sequence of the *i*,*i*+4 macrocyclized test peptide **12** without the N-terminal Cys and Met residues. After RCAM the alkyne macrocycle is dibrominated using $CuBr_2$. (b) HPLC traces of **12** in the open- (**10**, top), closed- (**11**, middle) and dibrominated conformation (**12**, bottom).The product peak is highlighted accordingly. (c) Mass spectra of the open- (top) and closed (middle) alkyne crosslink and the bibrominated olefin (bottom). The HPLC chromatograms are taken from crude reaction mixtures without any further purification.

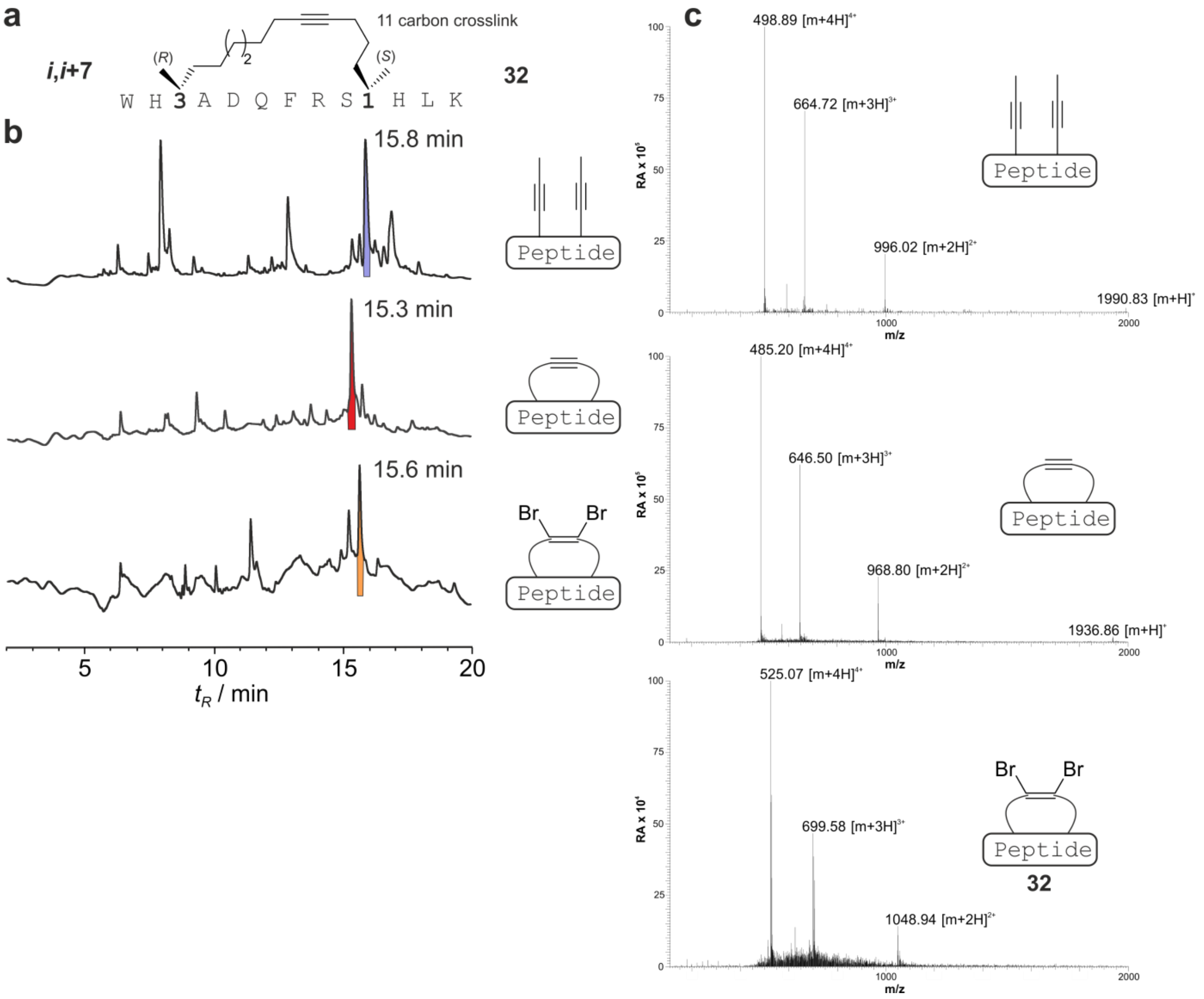

Supplementary Figure 8. (a) Sequence of the *i*,*i*+7 macrocyclized test peptide **32** without the N-terminal Cys and Met residues. After RCAM the alkyne macrocycle is dibrominated using $CuBr_2$. (b) HPLC traces of **32** in the open- (top), closed- (middle) and dibrominated conformation (bottom).The product peak is highlighted accordingly. (c) Mass spectra of the open- (top) and closed (middle) alkyne crosslink and the dibrominated olefin (bottom). The HPLC chromatograms are taken from crude reaction mixtures without any further purification.

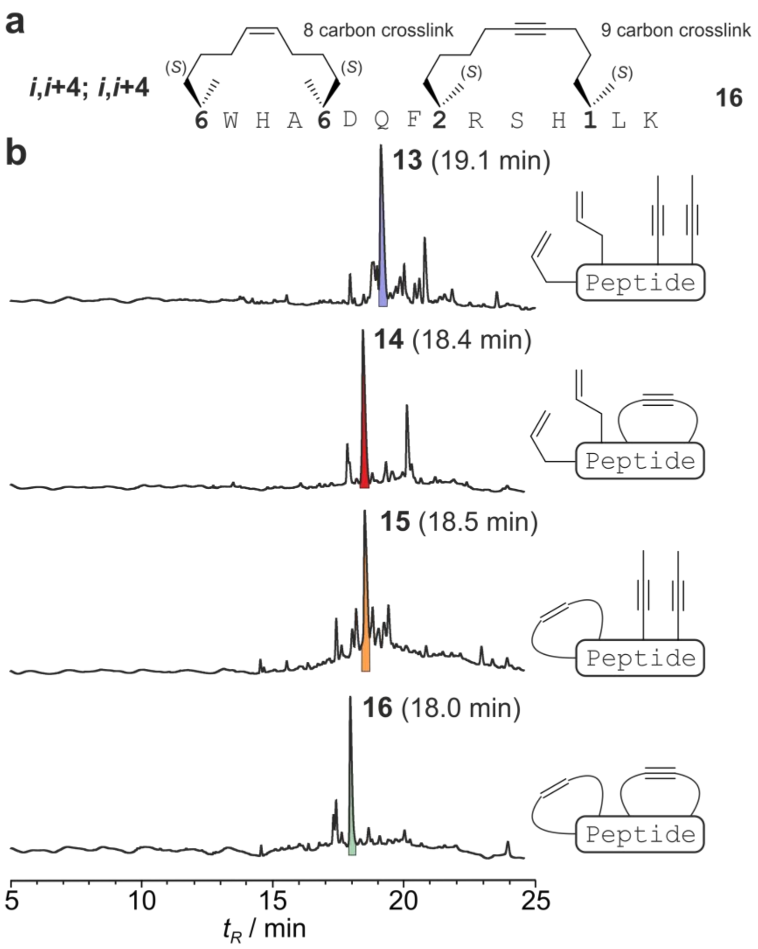

Supplementary Figure 9. (a) Sequence of the *i*,*i*+4; *i*,*i*+4 bicyclic peptide **16** without the N-terminal Cys and Met residues. The olefin and alkyne macrocycle can be closed without affecting the orthogonal functionalities. (b) HPLC traces of **16** in the open- (**13**, top), alkyne-closed- (**14**, second), olefin-closed- (**15**, third) and fully closed conformation (**16**, bottom).The product peak is highlighted accordingly. The HPLC chromatograms are taken from crude reaction mixtures without any further purification.

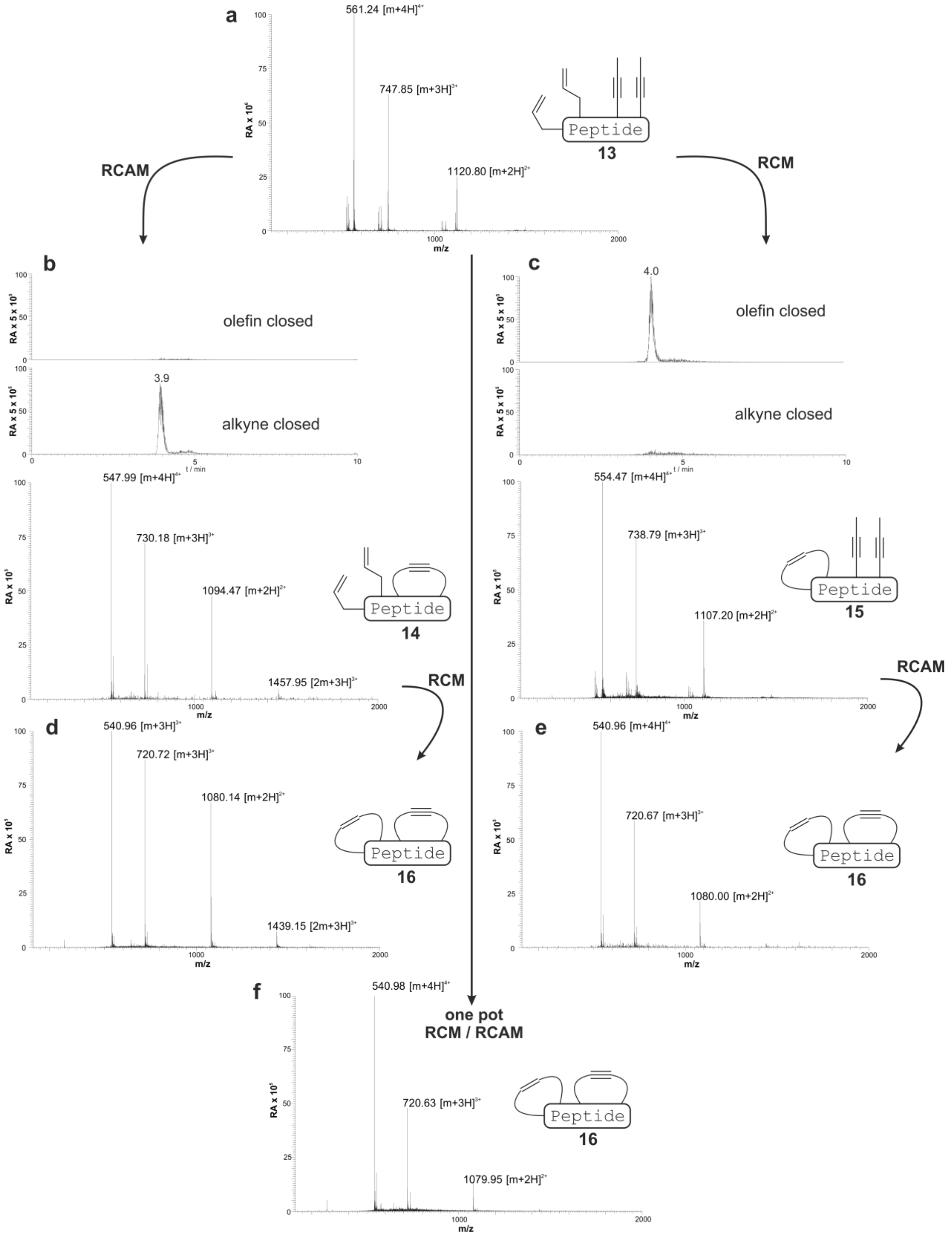

Supplementary Figure 10. Mass spectra of the *i*,*i*+4; *i*,*i*+4 bicyclic peptide **16**. (a) MS pattern of the fully open peptide precursor **13**. (b) HPLC-MS analysis of the alkyne macrocyclized intermediate (**14**, top). Only the alkyne macrocyclized intermediate and no olefin crosslinked intermediate can be detected. MS pattern of the alkyne macrocyclized intermediate (bottom). (c) HPLC-MS analysis of the olefin macrocyclized intermediate (**15**, top). Only the olefin macrocyclized intermediate and no alkyne crosslinked intermediate can be detected. MS pattern of the olefin macrocyclized intermediate (bottom). (d) MS pattern of the fully closed bicyclic alkyne/olefin peptide **16**. (e) MS pattern of the fully closed bicyclic olefin/alkyne peptide **16**. (f) MS pattern of the fully closed bicyclic one pot synthesis alkyne/olefin peptide **16**.

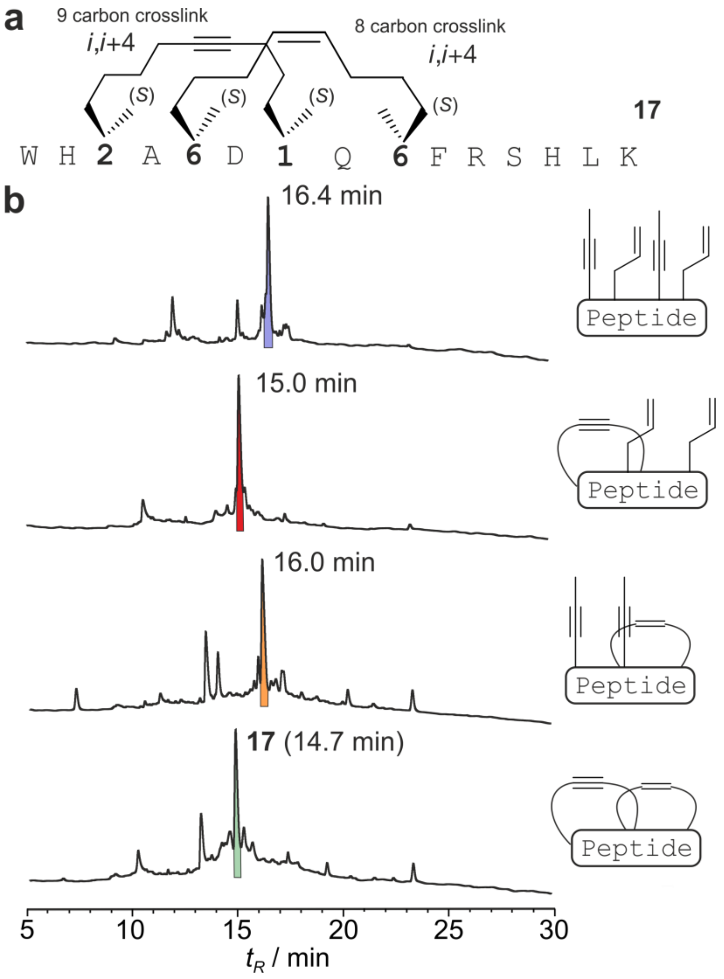

Supplementary Figure 11. (a) Sequence of bicyclic peptide **17** without the N-terminal Cys and Met residues. The olefin and alkyne macrocycle can be closed without affecting the orthogonal functionalities. (b) HPLC traces of **17** in the open- (top), alkyne-closed- (second), olefin-closed- (third)[a] and fully closed conformation (**17**, bottom).The product peak is highlighted accordingly. [a] Grubbs 1st gen. catalyst (2 mg/mL), dry toluene, 40°C, 2 x 1.5 h. The HPLC chromatograms are taken from crude reaction mixtures without any further purification.

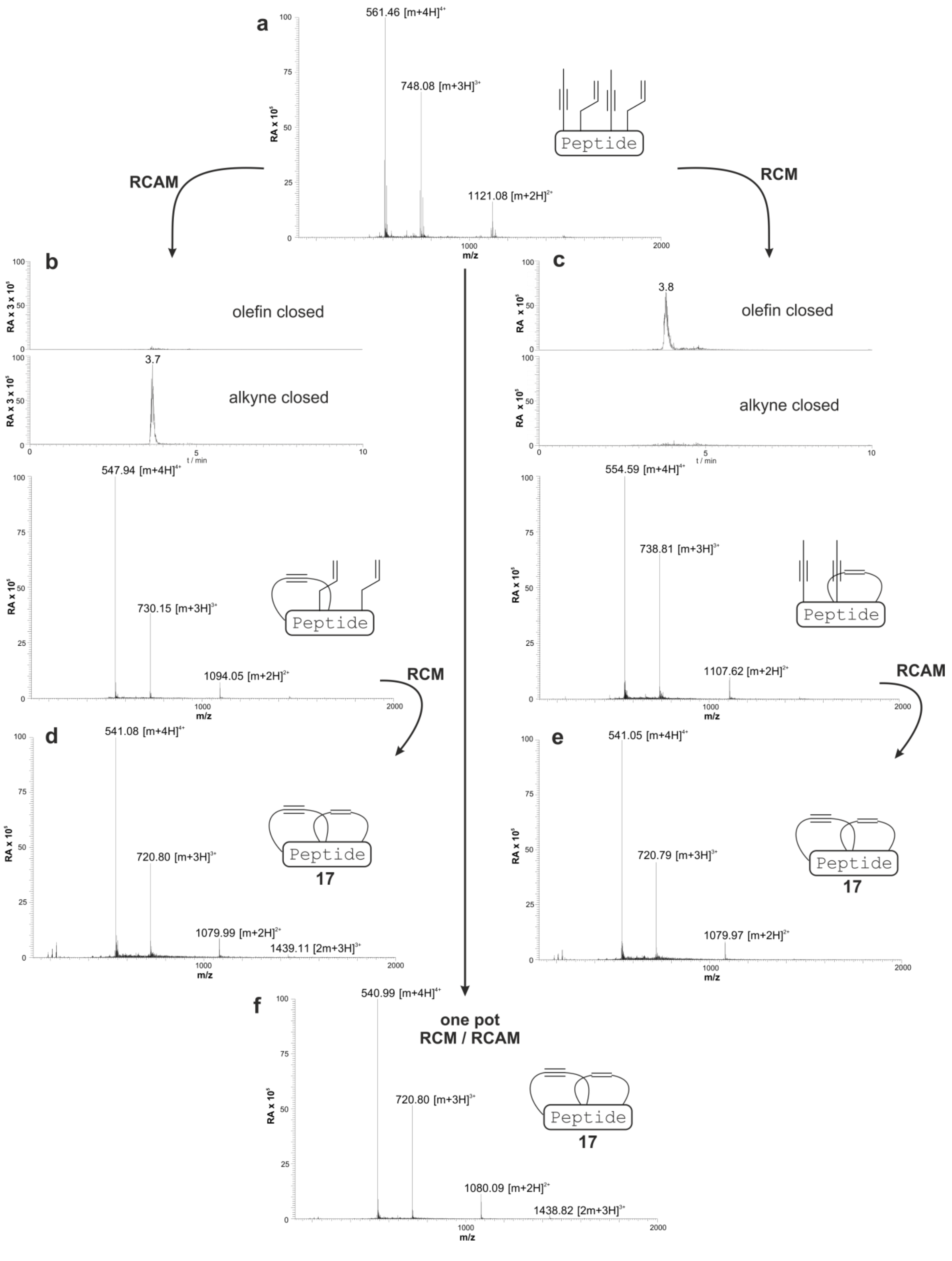

Supplementary Figure 12. Mass spectra of bicyclic peptide **17**. (a) MS pattern of the fully open peptide precursor. (b) HPLC-MS analysis of the alkyne macrocyclized intermediate (top). Only the alkyne macrocyclized intermediate and no olefin crosslinked intermediate can be detected. MS pattern of the alkyne macrocyclized intermediate (bottom). (c) HPLC-MS analysis of the olefin macrocyclized intermediate (top). Only the olefin macrocyclized intermediate and no alkyne crosslinked intermediate can be detected. MS pattern of the olefin macrocyclized intermediate (bottom). (d) MS pattern of the fully closed bicyclic alkyne/olefin peptide **17**. (e) MS pattern of the fully closed bicyclic olefin/alkyne peptide **17**. (f) MS pattern of the fully closed bicyclic one pot synthesis alkyne/olefin peptide **17**.

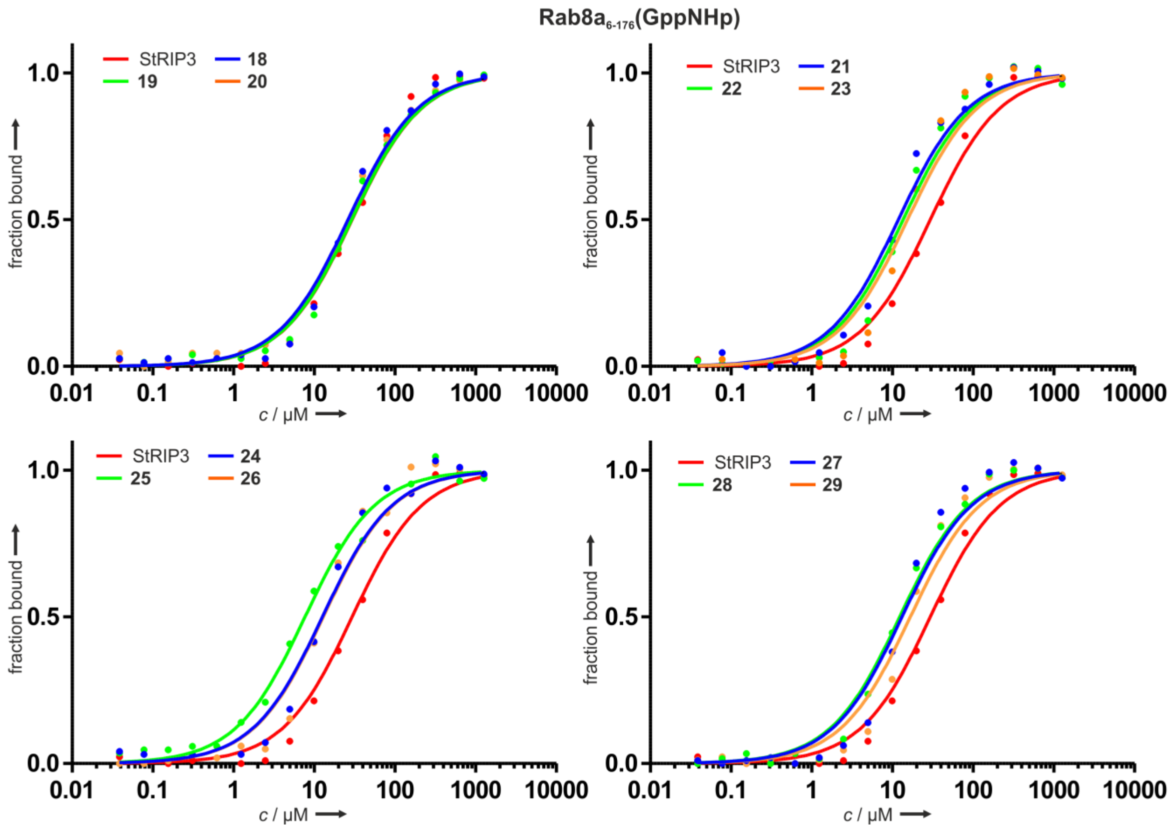

Supplementary Figure 13. Fitted FP data for all Rab8 binding peptides. (c_{max} [Rab8a$_{6\text{-}176}$(GppNHp)] = 1265 µM); (n = 1)

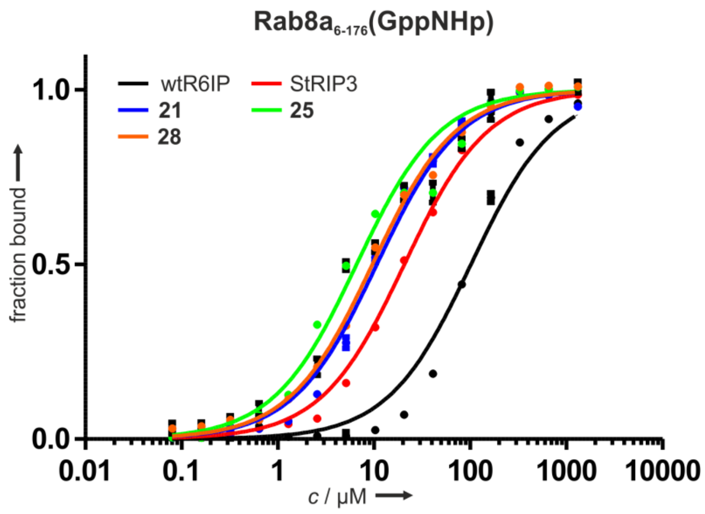

Supplementary Figure 14. Fitted FP data for the best Rab8 binding peptides (triplet measurements). (c_{max} [Rab8a$_{6\text{-}176}$(GppNHp)] = 1310 µM); (n = 3).

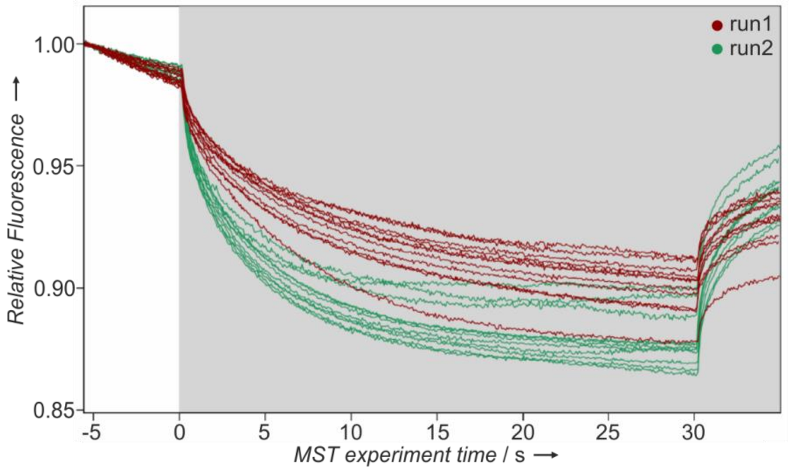

Supplementary Figure 15. MST curves for peptide **25**. The Results from MST experiments are summarized in Supplementary Table 5.

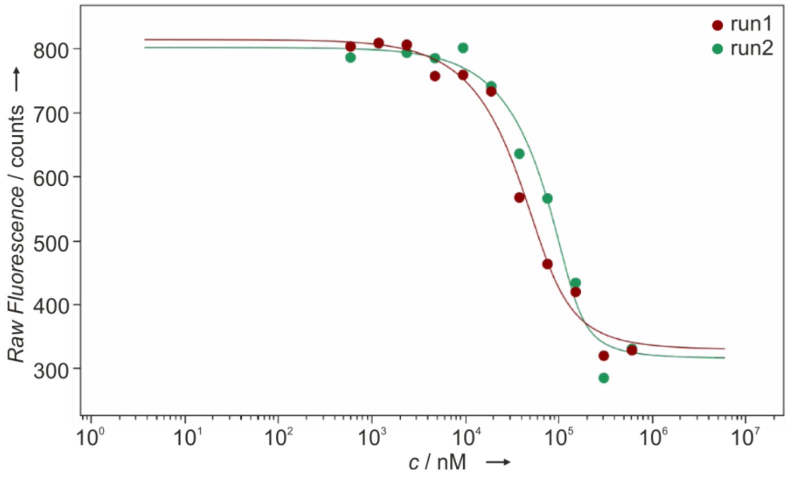

Supplementary Figure 16. Fitted initial fluorescence data for peptide **25**. (c_{max} [Rab8a$_{6\text{-}176}$(GppNHp)] = 605 µM). Calculated K_d values are summarized in Supplementary Table 5.

Supplementary Table 1. Optimization of the metathesis reaction for peptide **8**. The resin was dried in toluene over molecular sieve (5 Å) for four days while exchanging the solvent daily. The reactions were performed in 0.15 mL dry toluene and molecular sieve (5 Å) in a baked out Schlenk tube under argon for 3 h at 40° C.

Resin	Conversion [%]
Rink Amide Tentagel	Quant.[a]
Rink Amide MBHA	19[a]
Rink Amide ChemMatrix	36[a]
Rink Amide Tentagel	12[b]
Rink Amide MBHA	n.c. [b]
Rink Amide ChemMatrix	n.c. [b]

[a] 1.5 eq. of complex **5** according to resin loading
[b] 1.5 eq. of complex complex **33** according to resin loading
n.c.: no conversion

Supplementary Table 2. Detailed overview of all synthesized alkyne macrocyclic peptides based on the designed test sequence.

Entry	Peptide	Architecture	Carbon atoms crosslink	*N*-Term mod.	Sequence	HPLC (t_R, [min]) [a]	HRMS Calc.	HRMS (found)
**1**	**7**	*i*,*i*+3	**8**	**Fmoc**	C M W H A **4** D Q **1** F R S H L K	15.52	1064.50584	1064.51051 $[m+2H]^{2+}$
**2**	**30**	*i*,*i*+4	**8**	**Fmoc**	C M W H **1** A D Q **1** F R S H L K	14.94	1064.50584	1064.50663 $[m+2H]^{2+}$
**3**	**8**	*i*,*i*+4	**9**	**Fmoc**	C M W H **2** A D Q **1** F R S H L K	15.54	1071.51366	1071.51565 $[m+2H]^{2+}$
**4**	**9**	*i*,*i*+7	**11**	**Fmoc**	C M W H **3** A D Q F R S **1** H L K	15.58	1085.52931	1085.53116 $[m+2H]^{2+}$
**5**	**31**[b]	*i*,*i*+3	**8**	**Fmoc**	W H A **4** D Q **1** F R S H L K	16.39	1026.39934	1026.40084 $[m+2H]^{2+}$
**6**	**12**[b]	*i*,*i*+4	**9**	**Fmoc**	W H **2** A D Q **1** F R S H L K	16.61	1033.40717	1033.40909 $[m+2H]^{2+}$
**7**	**32**[b]	*i*,*i*+7	**11**	**Fmoc**	W H **3** A D Q F R S **1** H L K	15.60	1047.42282	1047.42485 $[m+2H]^{2+}$
**8**	**16**	*i*,*i*+4; *i*,*i*+4	**8,9**	**Fmoc**	***6*** W H A ***6*** D Q F **2** R S H 1 L K	18.03	720.05102	720.05153 $[m+3H]^{3+}$
**9**	**17**	*i*,*i*+4; *i*,*i*+4	**8,9**	**Fmoc**	W H **2** A **6** D **1** Q **6** F R S H L K	14.75	720.05102	720.05057 $[m+3H]^{3+}$

[a] Retention time of crude peptides by analytical HPLC (10-90% MeCN (0.1% TFA), 30 min)

[b] Dibrominated olefin

Supplementary Table 3. Detailed overview of all Rab8a binding peptides.

Entry	Peptide	*N*-Term mod. [a]	Sequence	Purity [%] [b]	HPLC (t_R, [min]) [c]	Yield [%] [d]	HRMS Calc.	HRMS (found)
**1a**	**wtR6IP**	**F**	D D E K E Q F L Y H L L S F N A V	84	16.58	43	1301.06757	1301.06883 $[m+2H]^{2+}$
**1b**		**Ac**		>99	13.95	70	1055.01801	1055.01900 $[m+2H]^{2+}$
**2a**	**StRIP3**	**F**	D D E ***6*** E Q F ***6*** Y H L L S F N A V	>99	20.61	60	1305.56213	1305.56853 $[m+2H]^{2+}$
**2b**		**Ac**		98	19.64	65	1059.51256	1059.51703 $[m+2H]^{2+}$
**3**	**18**	**F**	D D E **2** E Q F **2** Y H L L S F N A V	98	20.04	16	1318.56995	1318.56862 $[m+2H]^{2+}$
**4**	**19**	**F**	D D E **1** E Q F **2** Y H L L S F N A V	95	19.83	12	1311.56212	1311.56061 $[m+2H]^{2+}$
**5**	**20**	**F**	D D E **2** E Q F **1** Y H L L S F N A V	97	19.34	23	1311.56212	1311.56163 $[m+2H]^{2+}$
**6**	**21[e]**	**F**	D D E **2** E Q F **2** Y H L L S F N A V	96	19.04	15	1397.48829	1397.49138 $[m+2H]^{2+}$
**7**	**22[e]**	**F**	D D E **1** E Q F **2** Y H L L S F N A V	88	18.01	13	1390.48046	1390.48384 $[m+2H]^{2+}$
**8**	**23[e]**	**F**	D D E **2** E Q F **1** Y H L L S F N A V	97	17.52	21	1390.48046	1390.48449 $[m+2H]^{2+}$
**9**	**24**	**F**	D D E ***6*** E Q F ***6*** Y H L **2** S F N **2** V	95	19.82	15	901.39804	901.39757 $[m+2H]^{3+}$
**10a**	**25**	**F**	D D E ***6*** E Q F ***6*** Y H L **1** S F N **2** V	98	20.98	13	1344.58560	1344.58642 $[m+2H]^{2+}$
**10b**		**Ac**		98	16.70	20	1098.03212	1098.02466 $[m+2H]^{2+}$
**11**	**26**	**F**	D D E ***6*** E Q F ***6*** Y H L **2** S F N **1** V	98	20.21	25	1344.58560	1344.58476 $[m+2H]^{2+}$
**12**	**27**	**F**	D D E **2** E Q F **2** Y H L ***6*** S F N ***6*** V	97	19.88	30	901.39804	901.39818 $[m+2H]^{3+}$
**13**	**28**	**F**	D D E **1** E Q F **2** Y H L ***6*** S F N ***6*** V	93	18.62	12	1344.58560	1344.58396 $[m+2H]^{2+}$
**14**	**29**	**F**	D D E **2** E Q F **1** Y H L ***6*** S F N ***6*** V	98	18.96	54	1344.58560	1344.58768 $[m+2H]^{2+}$

[a] **F** = Fluorescein-O2OC-,Ac = Acetylated
[b] Calculated from UV-absorbance at 210 nm
[c] Retention time of purified peptides by analytical HPLC (10-90% MeCN (0.1% TFA), 30 min)
[d] Yield was determined according to Fmoc quantification after the 1st amino acid (Val) and quantification of the final peptide by UV absorption at 496 nm (fluorescein labeled peptides)
[e] Dibrominated olefin

Supplementary Table 4 Alkyne macrocyclized peptides and their binding affinities towards Rab8a$_{6-176}$(GppNHp). [a] singlet measurements (c_{max} [Rab8a$_{6-176}$(GppNHp)] = 1265 µM) [b] Dibrominated olefin.

900 905 910 915

D D E K E Q F L Y H L L S F N A V

Peptide	AA903	AA907	AA911	AA915	K_d [µM][a]
**StRIP3**	6	6	L	A	29.5
**18**	2	2	L	A	27.2
**19**	1	2	L	A	29.2
**20**	2	1	L	A	26.2
**21**[b]	2	2	L	A	11.8
**22**[b]	1	2	L	A	13.6
**23**[b]	2	1	L	A	15.4
**24**	6	6	2	2	12.6
**25**	6	6	1	2	8.1
**26**	6	6	2	1	12.9
**27**	2	2	6	6	13.1
**28**	1	2	6	6	12.3
**29**	2	1	6	6	16.7

Supplementary Table 5 Results from MST experiments (Supplementary Figure 16, 17). Initial fluorescence analysis of peptide **25**. Fluorescence data was fitted using the software Monolith Affinity Analysis (NanoTemper Technologies). Two individual runs were performed resulting in a K_d of 11 µM (average of two measurements).

Peptide **25**	K_d
run1	9 ± 8 µM
run2	13 ± 6 µM

Supplementary notes

Supplementary Note 1 List of abbreviations.

AcOH	Acetic acid
Ac_2O	Acetic anhydride
brine	Saturated NaCl (aqueous)
COMU	1-[(1-(Cyano-2-ethoxy-2-oxoethylidenaminooxy)-dimethylaminomorpholino)]-uronium-hexafluorphosphate
DCM	Dichloromethane
DIEA	Diisopropylethylamine
DMF	*N*,*N*-Dimethylformamide
EDT	1,2-Ethanedithiol
EDTA	Ethylenediaminetetraacetic acid
EA	Ethylacatate
ESI	Electrospray ionization
EtOH	Ethanol
FITC	Fluorescein isothiocyanate
Fmoc	Fluorenylmethoxycarbonyl
Fmoc-O2Oc-OH	Fmoc-8-amino-3,6-dioxaoctanoic acid
GppNHp	Guanosine-5'-(*β*, *γ*-imido)triphosphate
HCTU	*O*-(6-Chlorobenzotriazol-1-yl)-*N*,*N*,*N'*,*N'*-tetramethyluronium hexafluorophosphate
HEPES	2-[4-(2-hydroxyethyl)piperazin-1-yl]ethanesulfonic acid
HPLC	High-performance liquid chromatography
HRMS	High-resolution mass spectrometry
MeCN	Acetonitrile
MST	Microscale thermophoresis
n.c.	No conversion
NMP	*N*-Methyl-2-pyrrolidone
NMR	Nuclear magnetic resonance
Oxyma	Ethyl (hydroxyimino)cyanoacetate
PE	Petrol ether
RA	Relative Abundance
RCAM	Ring closing alkyne metathesis
RCM	Ring closing metathesis
SPPS	Solid-phase peptide synthesis
TBABr	Tetrabutyl ammonium bromide
TCEP	Tris(2-carboxyethyl)phosphine
TFA	Trifluroacetic acid
TIS	Triisopropylsilane

Supplementary Methods

Chemicals and instrumentation

Unless otherwise noted, chemicals were purchased from Sigma Aldrich, Merck, Okeanos, Roth or Alfa Aesar and were used without further purification. Protected Fmoc-amino acids and coupling reagents were purchased from Novabiochem and Iris Biotech GmbH. Building block **6** for hydrocarbon peptide stapling was purchased from Okeanos Tech. Co. LTD. All solvents were purchased from commercial suppliers and used without further purification. Analytical HPLC was performed using an Agilent 1100 Series with either a C18 HPLC column 3 µm (Macherey Nagel) or a C18 HPLC column 1.8 µm (Macherey Nagel). The system was run at a flow rate of 1.0 mL/min over 30 min using H_2O (0.1% TFA) and MeCN (0.1% TFA) as solvents. Linear gradients were run over varying periods of time. The efficiency of nucleotide exchange was monitored by analytical HPLC using 50 mM KH_2PO_4 (pH 7.0) and 10 mM TBABr and MeCN (0.1% TFA) as solvents. HPLC-MS analyses were performed with an Agilent 1100 Series connected to a Thermo LCQ Advantage mass spectrometer using a C18 HPLC column 3 µm (Macherey Nagel). The system was run at a flow rate of 1 mL/min over 15 min using H_2O (0.1% formic acid) and MeCN (0.1% formic acid) as eluents. Semi preparative HPLC was carried out on a Agilent 1100 Series using a SP125/10 Nuclear C18 Gravity 5 µm column (Macherey Nagel) at a flow rate of 6 mL/min. Linear gradients using H_2O (0.1% TFA) and MeCN (0.1% TFA) were run over varying periods of time. High resolution mass spectra were recorded on a QLT Orbitrap mass spectrometer coupled to an Acceka HPLC-System (HPLC column: Hypersyl GOLD, 50 mm x 1mm particle size 1.9 µm, ionization method: Electrospray Ionization). Automated Peptide synthesis was performed using a CEM-Discover microwave and a CEM-Liberty peptide synthesizer. Fluorescence polarization was measured with a Tecan Safire^2. Absorbance measurements were performed on a Tecan infinite M200 and Thermo scientific Nanodrop 2000c. ^{1}H- and ^{13}C-NMR spectra were recorded on a Varian Mercury VX 500 or 400 spectrometer at room temperature. NMR spectra were calibrated to the solvent signals $CDCl_3$ (7.26 and 77.16) or DMSO (2.50 and 39.52). MicroScale Thermophoresis (MST) curves were measured on a NanoTemper Technologies Monolith NT.115.

Peptide synthesis

General

Peptides were synthesized on solid-phase using the Fmoc-strategy and Rink Amide (MBHA) resin, Rink Amide NovaSyn TGR resin or ChemMatrix Rink Amide resin as solid support. Solvents and soluble reagents were removed by suction. Washings between coupling and deprotection were carried out in DMF and DCM using 1 mL solvent per 100 mg resin. Coupling efficiency was monitored by ESI-MS and/or HPLC analyses.

Fmoc group deprotection

The resin was swollen in DMF and treated with a solution of piperidine/DMF (20/80, v/v) for 2 x 5 min. Afterwards the resin was washed with DMF (3x), DCM (3x) and DMF (3x).

Amino acid coupling

Fmoc-Xaa-OH (4 eq.) was dissolved in freshly prepared solution of HCTU (3.9 eq., 0.5 M) with DIEA (8 eq.). Subsequently, this mixture was added to the resin and shaken for 30 min at room temperature. For coupling of the Alkyne building blocks (**1** – **4**), the building block **6** and the subsequent amino acid: Fmoc-Xaa-OH (4 eq.) was dissolved in DMF in the presence of COMU (3.9 eq.), Oxyma (3.9 eq.) and DIEA (8 eq.), added to the resin and shaken for 1 h at room temperature. Except coupling of the Alkyne building blocks **1** – **4** and the alkene building block **6**, all couplings were performed as double couplings. All equivalents are calculated based on theoretical loading of the resin as provided by the vendor.

N-Acetylation

For preparation of *N*-acetylated peptides and whenever a quantitative yield even after recoupling treatments was not achieved, the free *N*-terminal amino group was acetylated using a solution of Ac_2O/DIEA/DMF (1/1/8, v/v/v) for 2 x 10 min at room temperature.

Microwave peptide synthesis

Unmodified peptide sequences were synthesized with a microwave assisted peptide synthesizer. Removal of the Fmoc group was performed in piperidine/DMF (20/80, v/v), 1 min 30°C (intensity = 40 W) and 5 min 70 °C (intensity = 40 W). Coupling of amino acids was performed as double couplings in DMF, Fmoc-Xaa-OH (4 eq., 0.2 M), HCTU (3.9 eq., 0.5 M) and DIEA (8 eq., 0.2 M in NMP) for 10 min at 80°C (intensity = 20 W). Coupling of His- and Cys residues was performed as double couplings for 15 min at 60°C (intensity = 20 W).

Fluorescence labelling with FITC

Prior to fluorescence labelling with FITC a PEG-linker (Fmoc-O2Oc-OH) was coupled to the free *N*-terminus. A mixture of Fmoc-O2Oc-OH (5 eq.), COMU (4.9 eq.), Oxyma (4.9 eq.) and DIEA (10 eq.) in DMF was transferred to the resin and shaken at room temperature for 2 x 1 h. The resin was drained and washed with DMF (3x). The Fmoc

group was removed as described above and the resin was treated with FITC (5 eq.) and DIEA (10 eq.) for 16 h at room temperature under exclusion of light. Afterwards, the resin was washed with DMF (3x), DCM (3x) and dried to constant weight in vacuo.

Ring closing alkyne metathesis

The dried resin was transferred under argon into a baked out Schlenk tube and swollen and shrunken alternating in dry diethyl ether and dry toluene (3x each). Afterwards 0.5 mL of a solution of the alkyne metathesis complex **5** (2 mg mL^{-1}) in dry toluene was added and the reaction mixture was stirred at 40°C for 1.5 h. During the reaction time argon was bubbled through the reaction mixture to evaporate the 2-butyne. After addition of 0.5 mL of fresh complex **5** solution the mixture was stirred at 40° C for 1.5 h. The resin was filtered off, washed with toluene (3x), DCM (3x) and dried to constant weight.

Ring closing olefin metathesis

The dried resin was swollen in DCE for 15 min. A solution of Grubbs 1st generation catalyst (2 mg mL^{-1}) in DCE was added to the resin and reacted for 2 h at room temperature. During the reaction time argon was bubbled through the reaction mixture to remove ethene. The procedure was repeated twice and the resin was washed with DCE (3x), DCM (3x), DMF (3x).

One pot ring closing alkyne and olefin metathesis

The dried resin was transferred under argon into a baked out Schlenk tube and swollen and shrunk alternating in dry diethyl ether and dry toluene (3x each). Afterwards 0.5 mL of a solution of the alkyne metathesis complex **5** (2 mg mL^{-1}) and Grubbs 1st generation catalyst (2 mg mL^{-1}) in dry toluene was added and the reaction mixture stirred at 40° C for 1.5 h. During the reaction time argon was bubbled through the reaction mixture to evaporate the 2-butyne. After addition of 0.5 mL of fresh complex solution (alkyne complex **5** and Grubbs 1st generation catalyst) the mixture was stirred at 40° C for 1.5 h. The resin was filtered off, washed with toluene (3x), DCM (3x) and dried to constant weight.

Dibromination of alkyne macrocycles

The dried resin was swollen in dry MeCN for 15 min and treated with a mixture of $CuBr_2$ in dry MeCN (2 mg mL^{-1}) for 2 h. The reaction was performed in a Syringe

reactor and the procedure was repeated twice. Afterwards, the resin was washed with MeCN (3x), DMF (3x), DCM (3x).

Cleavage from the resin

The dry resin was treated with a solution of TFA/EDT/TIS/H_2O (94/1/2.5/2.5, v/v/v/v) 100 µL 10 mg^{-1} resin for 2 x 1 h and 1 x 5 min. The solvents were evaporated and the crude peptide was precipitated by the addition of cold diethyl ether. After centrifugation (10 min, 16.100 x *g*, 4°C) the supernatant was removed. The crude product was dissolved in H_2O/MeCN (2/1, v/v) and lyophilized. The crude peptides were purified by semi-preparative HPLC.

Fmoc quantification

A defined amount of dry resin was transferred into an Eppendorf cap and treated with 0.5 mL deprotection solution for 15 min. The UV absorption of the supernatant was determined at 305 nm and the occupation density calculated using Beer-Lambert law (ε = 7800 cm^{-1} M^{-1}).

Peptide quantification

The concentration of fluorescein labeled peptides was determined by UV absorption in 20 mM phosphate buffer (pH 8.5) at 496 nm (ε = 77.000 cm^{-1} M^{-1}). The concentration of acetylated peptides was determined gravimetrically or via UV absorption at 280 nm.

Biochemical methods

Protein expression and purification

Expression and purification of $Rab8a_{6\text{-}176}$ was performed analog to full-length Rab8a according to established protocols.[1,2]

Nucleotide exchange

Nucleotide exchange was performed according to previously established protocols.[2,3] Briefly, for nucleotide removal Mg^{2+} was removed by addition of a 5-fold excess of EDTA and reacted for 1 h at room temperature. The protein solution was desalted

using a PD-10 desalting column Sephadex G-25 DNA Grade (GE Healthcare) with elution buffer consisting of 20 mM HEPES (pH 7.5), 50 mM NaCl, 1 mM TCEP. After removal of Mg^{2+} the protein was diluted to 80 - 100 µM before addition of $ZnCl_2$ (500 µM) and $(NH_4)_2SO_4$ (200 mM). After addition of alkaline phosphatase (5 U mg^{-1} Rab protein) the mixture was incubated for 16 h at 4°C. For nucleotide exchange, the mixture contained a 5-fold excess of GppNHp during alkaline phosphatase incubation. Afterwards, the mixture was desalted using a PD-10 desalting column Sephadex G-25 DNA Grade (GE Healthcare) with elution buffer consisting of 25 mM HEPES (pH 7.5) 150 mM NaCl, 1 mM TCEP, 1 mM $MgCl_2$ and 1 µM GppNHp.

Fluorescence polarization assay for the determination of dissociation constants K_d

Rab8a$_{6\text{-}176}$ was serially diluted in a buffer containing 25 mM HEPES (pH 7.5), 150 mM NaCl, 1 mM $MgCl_2$, 1 mM TCEP, 0.01% tween 20 and 1 µM GppNHp (assay buffer), treated with 66 nM fluorescein-labeled peptides and incubated for 4 h at room temperature. Binding assays were performed in 384-well, small volume, black flat-bottom, non-binding plates (Greiner). Fluorescence polarization values (λ_{ex} = 470 nm, λ_{em} = 525 nm) were determined using a Safire II plate reader (Tecan) at room temperature. Initial studies for alkyne macrocyclized peptides were performed as single measurements. Final affinity measurements of a subset of peptides were performed in triplicates. After correction for changes in fluorescence intensity upon binding, the fluorescence anisotoropy data were converted into fraction bound (Supplementary equation 1) of the FITC- labeled peptide and fitted to a one-site binding model derived from the law of mass action using K_d as the only fitting parameter (Supplementary equation 2).[4] In case of incomplete binding due to the limited solubility of Rab proteins, anisotropy top-values were extrapolated and constrained as indicated below. Non-linear regression was performed in Prism 5.0 (Graphpad).[5]

$$fraction\ bound = \frac{A - A_{\mathrm{free}}}{A - A_{\mathrm{free}} + Q(A_{\mathrm{bound}} - A)} \qquad \text{(Supplementary equation 1)}$$

A: observed anisotropy; A_{free}: anisotropy of free fluorophore; A_{bound}: anisotoropy of bound fluorophore; Q: change in fluorescence intensity between free and bound state

$$fraction\ bound = \frac{K_{\mathrm{d}} + L_{\mathrm{T}} + c_{\mathrm{Rab}} - \sqrt{(K_{\mathrm{d}} + L_{\mathrm{T}} + c_{\mathrm{Rab}})^2 - 4 L_{\mathrm{T}}\, c_{\mathrm{Rab}}}}{2\, L_{\mathrm{T}}} \qquad \text{(Supplementary equation 2)}$$

K_d: dissociation constant; L_T: total concentration of labeled peptide; c_{Rab}: protein concentration

Microscale Thermophoresis (MST)

$Rab8a_{6\text{-}176}$ (GppNHp) was serially diluted in assay buffer and treated with 133 nM fluorescein-labeled peptide. After incubation for 4 h at room temperature the mixture was soaked into capillaries for microscale thermophoresis (MST) measurements. K_d values were calculated after initial fluorescene analysis of the obtained MST curves using the software Monolith Affinity Analysis (NanoTemper Technologies).

Competition fluorescence polarization assay

Acetylated peptides were serially diluted in assay buffer (1:1) and were incubated with a mixture of 60 nm fluorescein-labeled peptide and $Rab8a_{6\text{-}176}$ (GppNHp) (15 µM or 100 µM depending on K_d of the labeled peptide) for 1 h at room temperature. Fluorescence polarization was dertermined and half maximal inhibitory concentrations (IC_{50}) were calculated by nonlinear regression analysis of dose-response curves using Prism 5.0 software (GraphPad).[5]

Synthetic methods

Synthesis of the alkyne building blocks **1-4** was performed according to adapted protocols from *Y. N. Belokon et al.*[6] and *G. H. Bird et al.*[7] Schematic representation of the synthesis is summarized below.

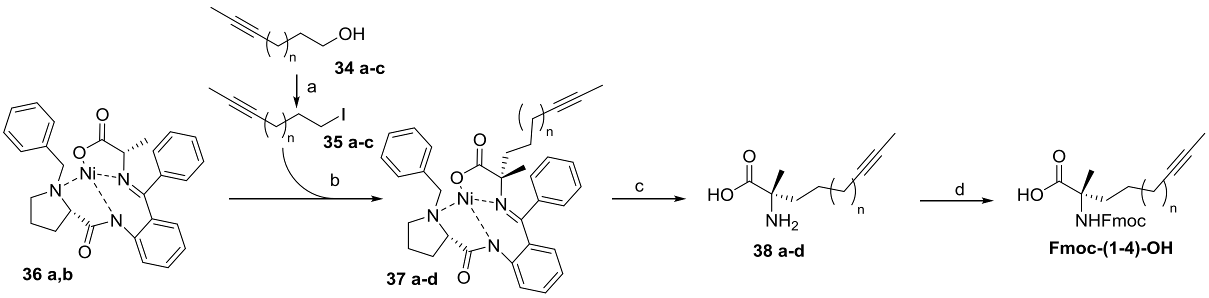

Synthesis of the alkyne building blocks. (a) PPh_3, I_2, Imidazol; THF, room temperature, 2 h; (b) KOH, 24 a-c; DMF, 0°C – room temperature, 2 h; (c) HCl, MeOH, reflux, 1 h; (d) Fmoc-OSu, Na_2CO_3, Dioxane/H_2O (1/1, v/v), room temperature, 7d. n = 1, 2, 4

Synthesis of Mo-complexes 5 and 33

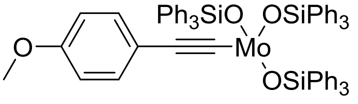

5

Chemical Formula: $C_{63}H_{52}MoO_4Si_3$
Exact Mass: 1054.22275
Molecular Weight: 1053.32000

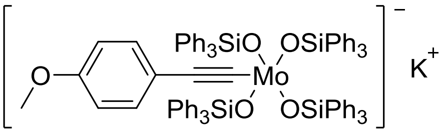

33

Chemical Formula: $C_{81}H_{67}KMoO_5Si_4$
Exact Mass: 1368.27567
Molecular Weight: 1367.82030

Mo-complexes for RCAM **5** and **33** were prepared according to previously established procedures.[8]

Synthesis of alkyne alcohols (34a-c)

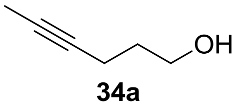

34a

Chemical Formula: $C_6H_{10}O$
Exact Mass: 98.07316
Molecular Weight: 98.14500

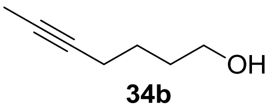

34b

Chemical Formula: $C_7H_{12}O$
Exact Mass: 112.08882
Molecular Weight: 112.17200

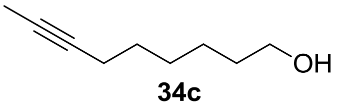

34c

Chemical Formula: $C_9H_{16}O$
Exact Mass: 140.12012
Molecular Weight: 140.22600

Synthesis of alkyne alcohols hex-4-yn-1-ol (**34a**), hept-5-yn-1-ol (**34b**) and non-7-yn-1-ol (**34c**), was carried out according to previously established protocols.[9]

Synthesis of iodo-alkynes (35a-c)

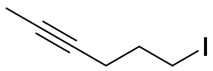

35a

Chemical Formula: C_6H_9I
Exact Mass: 207.97489
Molecular Weight: 208.04247

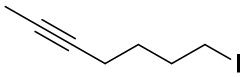

35b

Chemical Formula: $C_7H_{11}I$
Exact Mass: 221.99054
Molecular Weight: 222.06947

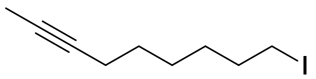

35c

Chemical Formula: $C_9H_{15}I$
Exact Mass: 250.02184
Molecular Weight: 250.12347

The alcohol **34a-c** was converted into the iodo-alkynes 6-iodohex-2-yne (**35a**), 7-iodohept-2-yne (**35b**) and 9-iodonon-2-yne (**35c**) following established protocols.[10]

35a

^{1}H NMR (500 MHz, $CDCl_3$): δ = 3.30 (t, J = 6.8 Hz, 2H), 2.29 – 2.23 (m, 2H), 1.95 (p, J = 6.7 Hz, 2H), 1.77 (t, J = 2.6 Hz, 3H). **^{13}C NMR** (126 MHz, $CDCl_3$): δ = 77.4, 77.1, 32.7, 19.9, 5.8, 3.6.

35b

^{1}H NMR (500 MHz, $CDCl_3$): δ = 3.20 (t, J = 7.0 Hz, 2H), 2.19 – 2.13 (m, 2H), 1.98 – 1.88 (m, 2H), 1.77 (t, J = 2.6 Hz, 3H), 1.62 – 1.52 (m, 2H). **^{13}C NMR** (126 MHz, $CDCl_3$): δ = 78.5, 76.3, 32.7, 29.9, 17.9, 6.5, 3.6.

35c

^{1}H NMR (500 MHz, $CDCl_3$): δ = 3.19 (t, J = 7.0 Hz, 2H), 2.16 – 2.08 (m, 2H), 1.87 – 1.79 (m, 2H), 1.77 (t, J = 2.5 Hz, 3H), 1.51 – 1.44 (m, 2H), 1.43 – 1.36 (m, 4H). **^{13}C NMR** (126 MHz, $CDCl_3$): δ = 79.2, 75.7, 33.6, 30.2, 28.9, 27.9, 18.8, 7.2, 3.6.

Synthesis of (*S*, *R*)-Ala-Ni(II)-BPB (36a,b)

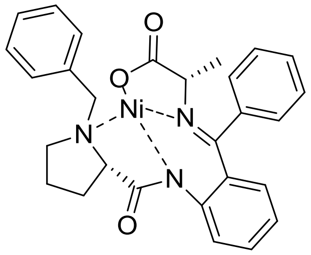

36a

Chemical Formula: $C_{28}H_{27}N_3NiO_3$
Exact Mass: 511.14059
Molecular Weight: 512.22568

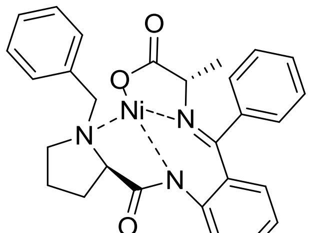

36b

Chemical Formula: $C_{28}H_{27}N_3NiO_3$
Exact Mass: 511.14059
Molecular Weight: 512.23540

Synthesis of the Ni-complexes **36a** and **36b** was carried out according to previously established protocols starting either from L- or D-Proline.[6,7,11]

36a:

^{1}H NMR (400 MHz, DMSO) δ = 8.4 (d, J = 7.5 Hz, 2H), 8.0 (d, J = 8.7 Hz, 1H), 7.6 – 7.5 (m, 4H), 7.4 (t, J = 7.7 Hz, 2H), 7.2 – 7.1 (m, 2H), 7.1 – 7.0 (m, 1H), 6.7 – 6.6 (m, 1H), 6.5 (dd, J = 8.2, 1.5 Hz, 1H), 4.1 (d, J = 12.3 Hz, 1H), 3.7 – 3.4 (m, 4H), 3.4 – 3.3 (m, 1H), 2.5 – 2.4 (m, 2H), 2.3 – 2.1 (m, 2H), 1.4 (d, J = 7.0 Hz, 3H). **^{13}C NMR** (101 MHz, DMSO): δ = 180.9, 179.0, 142.9, 135.5, 134.2, 133.2, 132.1, 131.9, 130.2, 129.6, 129.5, 129.2, 129.0, 128.3, 128.0, 126.4, 124.0, 120.7, 70.3, 66.6, 63.2, 58.2, 31.1, 24.4, 22.0. **HRMS**: calc. $[m+H]^+$ for $C_{28}H_{27}N_3NiO_3$ = 512.14787; found = 512.14789 $[m+H]^+$. **HPLC** (flow rate 1 mL/min, 10-90% MeCN (0.1% TFA), 13 min) = 7.93 min.

36b:

^{1}H NMR (600 MHz, DMSO) δ = 8.33 (d, J = 8.1 Hz, 2H), 7.93 (d, J = 8.7 Hz, 1H), 7.60 – 7.52 (m, 2H), 7.48 (m, 2H), 7.35 (dd, J = 10.8, 4.7 Hz, 2H), 7.13 (m, 2H), 7.05 (m, 1H), 6.64 (m, 1H), 6.49 (dd, J = 8.2, 1.6 Hz, 1H), 4.03 (d, J = 12.4 Hz, 1H), 3.58 – 3.47 (m, 4H), 3.35 – 3.30 (m, 1H), 2.47 – 2.38 (m, 2H), 2.23 – 2.10 (m, 2H), 1.42 (d, J = 7.1 Hz, 3H). **^{13}C NMR** (101 MHz, DMSO): δ = 180.2, 178.3, 169.4, 142.2, 134.7, 133.5, 132.5, 131.4, 131.6, 129.5, 128.9, 128.8, 128.5, 128.3, 127.6, 127.3, 125.7, 123.3, 120.0, 69.6, 65.9, 62.5, 57.5, 30.4, 23.7, 21.3. **HRMS**: calc. $[m+H]^+$ for $C_{28}H_{27}N_3NiO_3$ = 512.14787; found = 512.14810 $[m+H]^+$. **HPLC** (flow rate 1 mL/min, 10-90% MeCN (0.1% TFA), 13 min) = 8.12 min.

Synthesis of alkynated (*S*),(*R*)-Ala-Ni(II)-BPB (37a-d)

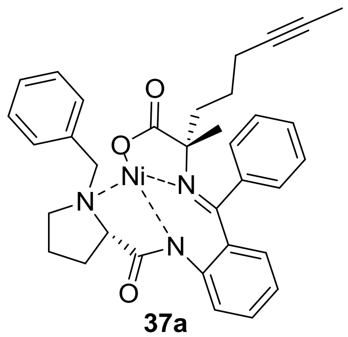

37a

Chemical Formula: $C_{34}H_{35}N_3NiO_3$
Exact Mass: 591.20319
Molecular Weight: 592.36540

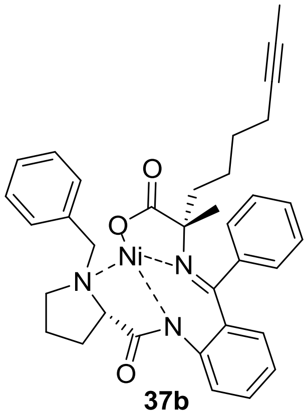

37b

Chemical Formula: $C_{35}H_{37}N_3NiO_3$
Exact Mass: 605.21884
Molecular Weight: 606.39240

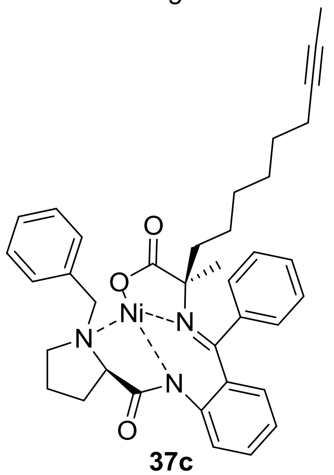

37c

Chemical Formula: $C_{37}H_{41}N_3NiO_3$
Exact Mass: 633.25014
Molecular Weight: 634.43314

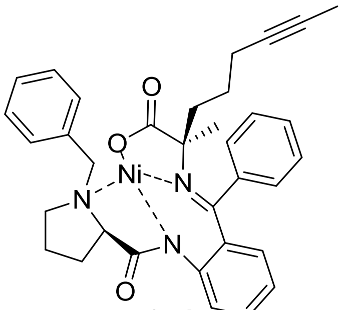

37d

Chemical Formula: $C_{34}H_{35}N_3NiO_3$
Exact Mass: 591.20319
Molecular Weight: 592.35340

To a solution of **36a,b** in 15 mL DMF in a baked out flask under argon, freshly ground KOH (5.0 eq.) was added and the reaction mixture stirred for 20 min at 0°C. After addition of iodo-alkynes (**30a-c**) (1.2 eq.) in 2 mL DMF, the mixture was stirred for 20 min at 0°C and another 2 h at room temperature. The reaction was quenched by pouring it onto chilled acetic acid (125 mL, 5%) and extracted with DCM (3 × 80 mL). The combined organic layers were washed with water (50 mL), brine and dried over $MgSO_4$. After co-evaporation with toluene the pure product was obtained as a red solid. Yields: **37a** = 98%; **37b** = 98%; **37c** = 99%; **37d** = 95%.

37a:

^{1}H NMR (500 MHz, DMSO) δ = 8.33 (d, J = 7.1 Hz, 2H), 7.92 – 7.86 (m, 1H), 7.54 – 7.47 (m, 3H), 7.46 – 7.38 (m, 3H), 7.24 (t, J = 7.5 Hz, 1H), 7.15 (d, J = 7.6 Hz, 1H), 7.11 – 7.04 (m, 1H), 6.68 – 6.61 (m, 1H), 6.57 (dd, J = 8.4, 1.5 Hz, 1H), 4.10 – 4.06 (m, 1H), 3.68 (d, J = 12.4 Hz, 1H), 3.52 (t, J = 8.3 Hz, 1H), 3.42 – 3.35 (m, 1H), 3.09 – 2.98 (m, 1H), 2.64 – 2.54 (m, 1H), 2.48 – 2.41 (m, 2H), 2.26 – 2.19 (m, 2H), 2.18 –

2.06 (m, 2H), 2.05 – 1.95 (m, 1H), 1.79 – 1.74 (m, 3H), 1.75 – 1.68 (m, 1H), 1.64 – 1.52 (m, 1H), 0.99 (s, J = 5.1 Hz, 3H). **^{13}C NMR** (126 MHz, DMSO) δ = 180.1, 180.0, 171.6, 141.8, 136.2, 134.7, 132.9, 131.5, 130.8, 130.6, 129.3, 128.5, 128.4, 127.8, 127.7, 127.2, 126.9, 123.4, 119.9, 78.8, 76.6, 76.3, 69.6, 63.0, 57.0, 39.9, 30.1, 28.7, 25.1, 22.7, 18.1, 3.1. **HRMS**: calc. $[m+H]^+$ for $C_{34}H_{36}N_3NiO_3$ = 592.21047; found = 592.21180 $[m+H]^+$. **HPLC** (flow rate 1 mL/min, 10-90% MeCN (0.1% TFA), 13 min) = 9.43 min.

37b:

^{1}H NMR (500 MHz, DMSO) δ = 8.32 (d, J = 7.1 Hz, 2H), 7.92 – 7.83 (m, 1H), 7.55 – 7.50 (m, 3H), 7.47 – 7.39 (m, 3H), 7.25 (t, J = 7.4 Hz, 1H), 7.13 (d, J = 8.3 Hz, 1H), 7.09 – 7.04 (m, 1H), 6.67 – 6.54 (m, 2H), 4.06 (d, J = 12.4 Hz, 1H), 3.70 (d, J = 12.4 Hz, 1H), 3.54 – 3.45 (m, 1H), 3.37 (dd, J = 18.7, 9.7 Hz, 1H), 3.12 – 2.99 (m, 1H), 2.55 – 2.50 (m, 2H), 2.48 – 2.41 (m, 1H), 2.26 – 2.20 (m, 2H), 2.17 – 2.06 (m, 2H), 1.99 – 1.92 (m, 1H), 1.65 – 1.58 (m, 3H), 1.50 – 1.37 (m, 4H), 1.06 (s, J = 8.9 Hz, 3H). **^{13}C NMR** (126 MHz, DMSO) δ = 180.5, 179.9, 171.7, 141.7, 136.3, 134.7, 132.7, 131.5, 131.3, 130.7, 130.4, 129.3, 128.5, 128.4, 127.9, 127.8, 127.1, 126.9, 123.5, 119.9, 79.1, 76.7, 76.1, 69.7, 62.9, 56.9, 40.0, 30.2, 29.0, 28.4, 24.4, 22.7, 18.1, 2.9. **HRMS**: calc. $[m+H]^+$ for $C_{35}H_{38}N_3NiO_3$ = 606.22613; found = 606.22693 $[m+H]^+$. **HPLC** (flow rate 1 mL/min, 10-90% MeCN (0.1% TFA), 13 min) = 9.61 min.

37c:

^{1}H NMR (500 MHz, DMSO) δ = 8.32 (d, J = 7.4 Hz, 2H), 7.86 (d, J = 8.6 Hz, 1H), 7.51 (s, 3H), 7.42 (t, J = 7.6 Hz, 3H), 7.25 (t, J = 7.4 Hz, 1H), 7.14 – 7.05 (m, 2H), 6.64 (t, J = 7.6 Hz, 1H), 6.57 (d, J = 8.4 Hz, 1H), 4.07 (d, J = 12.4 Hz, 1H), 3.69 (d, J = 12.4 Hz, 1H), 3.51 (dd, J = 9.7, 6.8 Hz, 1H), 3.40 – 3.34 (m, 1H), 3.04 (dd, J = 19.0, 9.0 Hz, 1H), 2.48 – 2.42 (m, 2H), 2.40 – 2.32 (m, 1H), 2.18 – 2.08 (m, 4H), 1.88 – 1.76 (m, 1H), 1.72 (s, 3H), 1.45 (m, 4H), 1.43 – 1.19 (m, 4H), 1.05 (s, 3H). **^{13}C NMR** (126 MHz, DMSO) δ = 180.6, 180.0, 171.6, 141.7, 136.3, 134.7, 132.8, 131.5, 130.7, 130.5, 129.3, 128.5, 128.5, 127.9, 127.8, 127.1, 126.9, 123.6, 120.0, 79.3, 76.8, 75.7, 69.6, 62.9, 57.0, 39.8, 30.2, 29.0, 28.6, 28.4, 28.3, 25.3, 22.8, 18.0, 3.1. **HRMS**: calc. $[m+H]^+$ for $C_{37}H_{42}N_3NiO_3$ = 634.25742; found = 634.25868 $[m+H]^+$. **HPLC** (flow rate 1 mL/min, 10-90% MeCN (0.1% TFA), 11 min) = 6.96 min.

37d:

^{1}H NMR (500 MHz, DMSO) δ = 8.33 (d, J = 7.1 Hz, 2H), 7.90 – 7.86 (m, 1H), 7.56 – 7.48 (m, 3H), 7.46 – 7.39 (m, 3H), 7.24 (t, J = 7.5 Hz, 1H), 7.15 (d, J = 7.7 Hz, 1H), 7.11 – 7.06 (m, 1H), 6.67 – 6.61 (m, 1H), 6.61 – 6.55 (m, 1H), 4.07 (d, J = 12.4 Hz, 1H), 3.68 (d, J = 12.4 Hz, 1H), 3.57 – 3.49 (m, 1H), 3.41 – 3.33 (m, 1H), 3.10 – 2.97 (m, 1H), 2.67 – 2.53 (m, 1H), 2.49 – 2.40 (m, 2H), 2.21 (s, J = 2.1 Hz, 2H), 2.18 –

2.05 (m, 2H), 2.02 – 1.95 (m, 1H), 1.76 (t, J = 2.4 Hz, 3H), 1.74 – 1.68 (m, 1H), 1.63 – 1.52 (m, 1H), 0.99 (s, 3H). **^{13}C NMR** (126 MHz, DMSO) δ = 180.1, 180.0, 171.6, 141.8, 136.2, 134.7, 132.9, 131.5, 130.8, 130.6, 129.3, 128.5, 128.4, 127.8, 127.7, 127.2, 126.9, 123.4, 119.9, 78.8, 76.6, 76.3, 69.6, 63.0, 57.0, 39.9, 30.1, 28.7, 25.1, 22.7, 18.1, 3.1. **HRMS**: calc. $[m+H]^+$ for $C_{34}H_{36}N_3NiO_3$ = 592.21047; found = 592.21180 $[m+H]^+$. **HPLC** (flow rate 1 mL/min, 10-90% MeCN (0.1% TFA), 13 min) = 9.16 min.

Synthesis of unprotected α-methyl-α-alkinyl amino acids (38a-d)

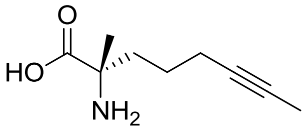

38a

Chemical Formula: $C_9H_{15}NO_2$
Exact Mass: 169.11028
Molecular Weight: 169.22400

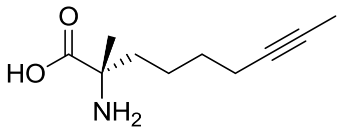

38b

Chemical Formula: $C_{10}H_{17}NO_2$
Exact Mass: 183.12593
Molecular Weight: 183.25100

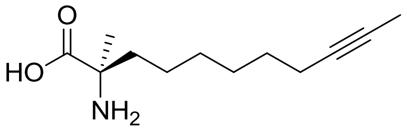

38c

Chemical Formula: $C_{12}H_{21}NO_2$
Exact Mass: 211.15723
Molecular Weight: 211.30064

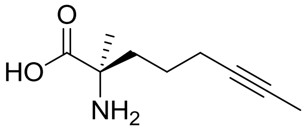

38d

Chemical Formula: $C_9H_{15}NO_2$
Exact Mass: 169.11028
Molecular Weight: 169.22090

To a solution of **37a-d** in MeOH (40 mL), conc. hydrochloric acid (10 eq.) was added and the reaction mixture refluxed at 80 °C for 1 h. The reaction mixture was allowed to cool to room temperature and concentrated *in vacuo*. After addition of 20 mL water (20 mL) the aqueous layer was extracted with DCM (3 × 20 mL). The combined organic layers were washed with brine, dried over $MgSO_4$ and concentrated under reduced pressure. Recovered BPB was purified by precipitation as hydrochloric salt from acetone solution.[12] The aqueous layer was dried by lyophilization and the crude unprotected α-methyl-α-alkinyl amino acid was used without any further purification.

Synthesis of Fmoc-protected α-methyl-α-alkinyl amino acids (1-4)

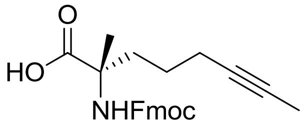

Fmoc-1-OH

Chemical Formula: $C_{24}H_{25}NO_4$
Exact Mass: 391.17836
Molecular Weight: 391.46700

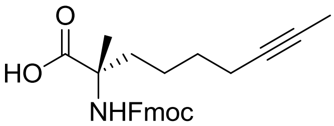

Fmoc-2-OH

Chemical Formula: $C_{25}H_{27}NO_4$
Exact Mass: 405.19401
Molecular Weight: 405.49400

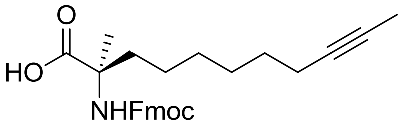

Fmoc-3-OH

Chemical Formula: $C_{27}H_{31}NO_4$
Exact Mass: 433.22531
Molecular Weight: 433.53934

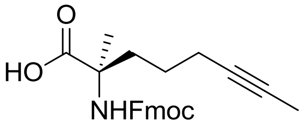

Fmoc-4-OH

Chemical Formula: $C_{24}H_{25}NO_4$
Exact Mass: 391.17836
Molecular Weight: 391.45960

To a solution of the crude unprotected α-methyl-α-alkynyl amino acid in H_2O/dioxane (40 mL, 1/1, v/v), Na_2CO_3 (4 eq.) and Fmoc-OSu (1.2 eq.) were added and stirred at room temperature for 7 d. The reaction was monitored using HPLC-MS analysis, daily and subsequently fresh Fmoc-Osu (0.5 eq.) was added. After addition of water (100 mL) the pH of the aqueous layer was set to 2-4 using aqueous hydrochloric acid and the aqueous layer was extracted with ethylacatate (3 x 100 mL). The combined organic layers were washed with brine, dried over $MgSO_4$ and concentrated under reduced pressure. The crude product was purified via column chromatography (R_f = 0.45, PE:EA 1:1; 0.1% AcOH) and obtained as a pale yellow solid. Yields: **1a** = 98%; **2b** = 97%; **3c** = 86%; **4d** = 90%.

Fmoc-1-OH

^{1}H NMR (500 MHz, DMSO) δ = 12.38 (s,1H), 7.89 (d, J = 7.5 Hz, 2H), 7.71 (d, J = 7.4 Hz, 2H), 7.43 (s, 1H), 7.41 (d, J = 7.3 Hz, 2H), 7.33 (t, J = 7.4 Hz, 2H), 4.31 – 4.22 (m, 2H), 4.22 – 4.16 (m, 1H), 2.14 – 2.01 (m, 2H), 1.88 – 1.79 (m, 1H), 1.79 – 1.73 (m, 1H), 1.72 (s, 3H), 1.45 – 1.33 (m, 2H), 1.32 (s, 3H). **^{13}C NMR** (126 MHz, DMSO) δ = 175.2, 154.6, 143.8, 140.7, 127.6, 127.0, 125.2, 120.0, 79.1, 75.8, 65.2, 58.1, 46.7, 36.0, 23.3, 22.5, 18.3, 3.1. **HRMS**: calc. $[m+H]^+$ for $C_{24}H_{26}NO_4$ = 392.18563 ; found = 392.18459 $[m+H]^+$, 414.16581 $[m+Na]^+$. **HPLC**: (flow rate 1 mL/min, 10-90% MeCN (0.1% TFA), 13 min) = 9.73 min.

Fmoc-2-OH

^{1}H NMR (500 MHz, DMSO) δ = 12.37 (s, 1H), 7.89 (d, J = 7.5 Hz, 2H), 7.72 (d, J = 7.4 Hz, 2H), 7.42 (d, J = 7.4 Hz, 2H), 7.36 (d, J = 16.1 Hz, 1H), 7.33 (t, J = 7.4 Hz,

2H), 4.34 – 4.22 (m, 2H), 4.22 – 4.18 (m, 1H), 2.17 – 2.03 (m, 2H), 1.76 (s, 1H), 1.69 (t, J = 2.4 Hz, 3H), 1.68 – 1.59 (m, 1H), 1.45 – 1.35 (m, 2H), 1.33 (s, 3H), 1.30 – 1.19 (m, 2H). **^{13}C NMR** (126 MHz, DMSO) δ = 175.3, 154.7, 143.8, 140.7, 127.6, 127.0, 125.2, 120.0, 79.1, 75.7, 65.2, 58.2, 46.7, 36.2, 28.7, 22.6, 22.4, 18.0, 3.1. **HRMS**: calc. $[m+H]^+$ for $C_{25}H_{28}NO_4$ = 406.20128 ; found = 406.20119 $[m+H]^+$, 428.18273 $[m+Na]^+$. **HPLC**: (flow rate 1 mL/min, 10-90% MeCN (0.1% TFA), 13 min) = 10.12 min.

Fmoc-3-OH

^{1}H NMR (500 MHz, DMSO) δ = 12.32 (s, 1H), 7.89 (d, J = 7.5 Hz, 2H), 7.72 (d, J = 7.4 Hz, 2H), 7.41 (t, J = 7.4 Hz, 2H), 7.37 (s, 1H), 7.35 – 7.30 (m, 2H), 4.32 – 4.22 (m, 2H), 4.22 – 4.17 (m, 1H), 2.11 – 2.03 (m, 2H), 1.75 (s, 1H), 1.71 (t, J = 2.4 Hz, 3H), 1.69 – 1.61 (m, 1H), 1.43 – 1.35 (m, 2H), 1.33 (s, 3H), 1.25 (s, 2H), 1.23 (m, 4H). **^{13}C NMR** (126 MHz, DMSO) δ = 175.4, 154.7, 143.8, 140.7, 127.6, 127.0, 125.2, 120.0, 79.3, 75.6, 65.2, 58.3, 46.7, 36.6, 28.7, 28.4, 28.1, 23.1, 22.3, 18.0, 3.1. **HRMS**: calc. $[m+H]^+$ for $C_{27}H_{31}NO_4$ = 434.23258 ; found = 434.23458 $[m+H]^+$, 456.21638 $[m+Na]^+$. **HPLC**: (flow rate 1 mL/min, 10-90% MeCN (0.1% TFA), 11 min) = 7.18 min.

Fmoc-4-OH

^{1}H NMR (500 MHz, DMSO) δ = 12.37 (s, 1H), 7.89 (d, J = 7.5 Hz, 2H), 7.72 (d, J = 7.4 Hz, 2H), 7.43 (s, 1H), 7.42 (t, J = 7.6 Hz, 2H), 7.33 (t, J = 7.4 Hz, 2H), 4.29 – 4.22 (m, 2H), 4.22 – 4.17 (m, 1H), 2.14 – 2.01 (m, 2H), 1.89 – 1.79 (m, 1H), 1.76 (s, J = 11.9 Hz, 1H), 1.72 (s, 3H), 1.43 – 1.34 (m, 2H), 1.32 (s, 3H). **^{13}C NMR** (126 MHz, DMSO) δ = 175.2, 154.7, 143.8, 140.7, 127.6, 127.0, 125.3, 120.0, 79.0, 75.9, 65.2, 58.1, 46.7, 36.0, 23.2, 22.4, 18.3, 3.1. **HRMS**: calc. $[m+H]^+$ for $C_{24}H_{26}NO_4$ = 392.18563 ; found = 392.18504 $[m+H]^+$, 414.16645 $[m+Na]^+$. **HPLC**: (flow rate 1 mL/min, 10-90% MeCN (0.1% TFA), 13 min) = 9.60 min.

Supplementary References

1. Bleimling, N. Alexandrov, K. Goody, R. & Itzen, A. Chaperone-assisted production of active human Rab8A GTPase in Escherichia coli. *Protein Expr. Purif.* **65,** 190–195 (2009).
2. Hou, X. *et al.* A structural basis for Lowe syndrome caused by mutations in the Rab-binding domain of OCRL1. *EMBO J.* **30,** 1659–1670 (2011).
3. Simon, I. Zerial, M. & Goody, R. S. Kinetics of Interaction of Rab5 and Rab7 with Nucleotides and Magnesium Ions. *J. Biol. Chem.* **271,** 20470–20478 (1996).
4. Huang, X. & Aulabaugh, A. in *High Throughput Screening,* edited by W. P. Janzen & P. Bernasconi (Humana Press, Totowa, NJ, 2009), pp. 127–143.
5. Motulsky, H. & Christopoulos, A. *Fitting models to biological data using linear and nonlinear regression. A practical guide to curve fitting* (Oxford University Press, Oxford, New York, 2004).
6. Belokon', Y. N. Tararov, V. I. Maleev, V. I. Savel'eva, T. F. & Ryzhov, M. G. Improved procedures for the synthesis of (S)-2-[N-(N′-benzylprolyl)amino]benzophenone (BPB) and Ni(II) complexes of Schiff's bases derived from BPB and amino acids. *Tetrahedron: Asymmetry* **9,** 4249–4252 (1998).
7. Bird, G. H. Crannell, C. W. & Walensky, L. D. Chemical Synthesis of Hydrocarbon-Stapled Peptides for Protein Interaction Research and Therapeutic Targeting. *Curr. Protoc. Chem. Biol.* **3,** 99–117 (2011).
8. Heppekausen, J. *et al.* Optimized synthesis, structural investigations, ligand tuning and synthetic evaluation of silyloxy-based alkyne metathesis catalysts. *Chem. Eur. J.* **18,** 10281–10299 (2012).
9. Fürstner, A. Guth, O. Rumbo, A. & Seidel, G. Ring Closing Alkyne Metathesis. Comparative Investigation of Two Different Catalyst Systems and Application to the Stereoselective Synthesis of Olfactory Lactones, Azamacrolides, and the Macrocyclic Perimeter of the Marine Alkaloid Nakadomarin A. *J. Am. Chem. Soc.* **121,** 11108–11113 (1999).
10. Bindl, M. Jean, L. Herrmann, J. Müller, R. & Fürstner, A. Preparation, Modification, and Evaluation of Cruentaren A and Analogues. *Chem. Eur. J.* **15,** 12310–12319 (2009).
11. Spiegel, J. *et al.* Direct targeting of Rab-GTPase-effector interactions. *Angew. Chem. Int. Ed.* **53,** 2498–2503 (2014).
12. Ueki, H. *et al.* Improved Synthesis of Proline-Derived Ni(II) Complexes of Glycine: Versatile Chiral Equivalents of Nucleophilic Glycine for General Asymmetric Synthesis of α-Amino Acids. *J. Org. Chem.* **68,** 7104–7107 (2003).
